# Multi-omic stable isotope probing reveals *myo*-inositol metabolism by anaerobic gut bacteria in laying hens

**DOI:** 10.1093/ismeco/ycag257

**Published:** 2026-09-04

**Authors:** Harshita Naithani, Johannes Günther, Timur Yergaliyev, Amélia Camarinha-Silva, Jana Seifert

**Affiliations:** Institute of Animal Science, University of Hohenheim, 70599 Stuttgart, Germany; HoLMiR - Hohenheim Center for Livestock Microbiome Research, University of Hohenheim, 70599 Stuttgart, Germany; Core Facility Spectroscopy, University of Hohenheim, 70599 Stuttgart, Germany; Institute of Animal Science, University of Hohenheim, 70599 Stuttgart, Germany; HoLMiR - Hohenheim Center for Livestock Microbiome Research, University of Hohenheim, 70599 Stuttgart, Germany; Institute of Animal Science, University of Hohenheim, 70599 Stuttgart, Germany; HoLMiR - Hohenheim Center for Livestock Microbiome Research, University of Hohenheim, 70599 Stuttgart, Germany; Institute of Animal Science, University of Hohenheim, 70599 Stuttgart, Germany; HoLMiR - Hohenheim Center for Livestock Microbiome Research, University of Hohenheim, 70599 Stuttgart, Germany

**Keywords:** *myo*-inositol, laying hen, metaproteomic-SIP, ^13^carbon NMR, anaerobic metabolization

## Abstract

*Myo*-inositol (MI) is a dietary cyclitol and phytate hydrolysis product whose microbial fate in poultry is poorly understood. We combined ^13^C-labelled MI, metaproteomic stable-isotope probing (SIP), untargeted metaproteomics, and ^13^C-nuclear magnetic resonance metabolomics to map MI utilization by anaerobic bacterial enrichment cultures established from ileal and caecal digesta of laying hens. This integrative approach enables activity-resolved tracking of carbon flow from substrate to proteins and fermentation end products within a complex anaerobic community. We reconstructed, for the first time, a community-resolved pathway of MI degradation in the chicken gut, linking labelled metabolites to taxon-specific protein synthesis. MI metabolism was spatially structured, with rapid and sustained utilization in the caeca and delayed, host-variable activity in the ileum. Metaproteomic-SIP identified *Megamonas* as a dominant MI-assimilating genus in the caeca and implicated *Gallibacterium* as a previously unrecognized contributor in the ileum. Despite minimal shifts in community composition inferred from metaproteomics, protein expression profiles changed markedly, indicating that functional responses to MI occurred independently of taxonomic restructuring. Pathway reconstruction, supported by labelled and unlabelled proteins and labelled metabolite dynamics, revealed conversion of MI into central metabolic intermediates and short-chain fatty acids (SCFAs), providing a mechanistic framework for microbial MI utilization. Together, these findings establish stable isotope probing multi-omics as a powerful strategy for reconstructing metabolic pathways in complex microbiomes and demonstrate the metabolic potential of chicken gut-derived microbial communities to utilize MI under controlled anaerobic enrichment conditions.

## Graphical Abstract

**Graphical Abstract:**
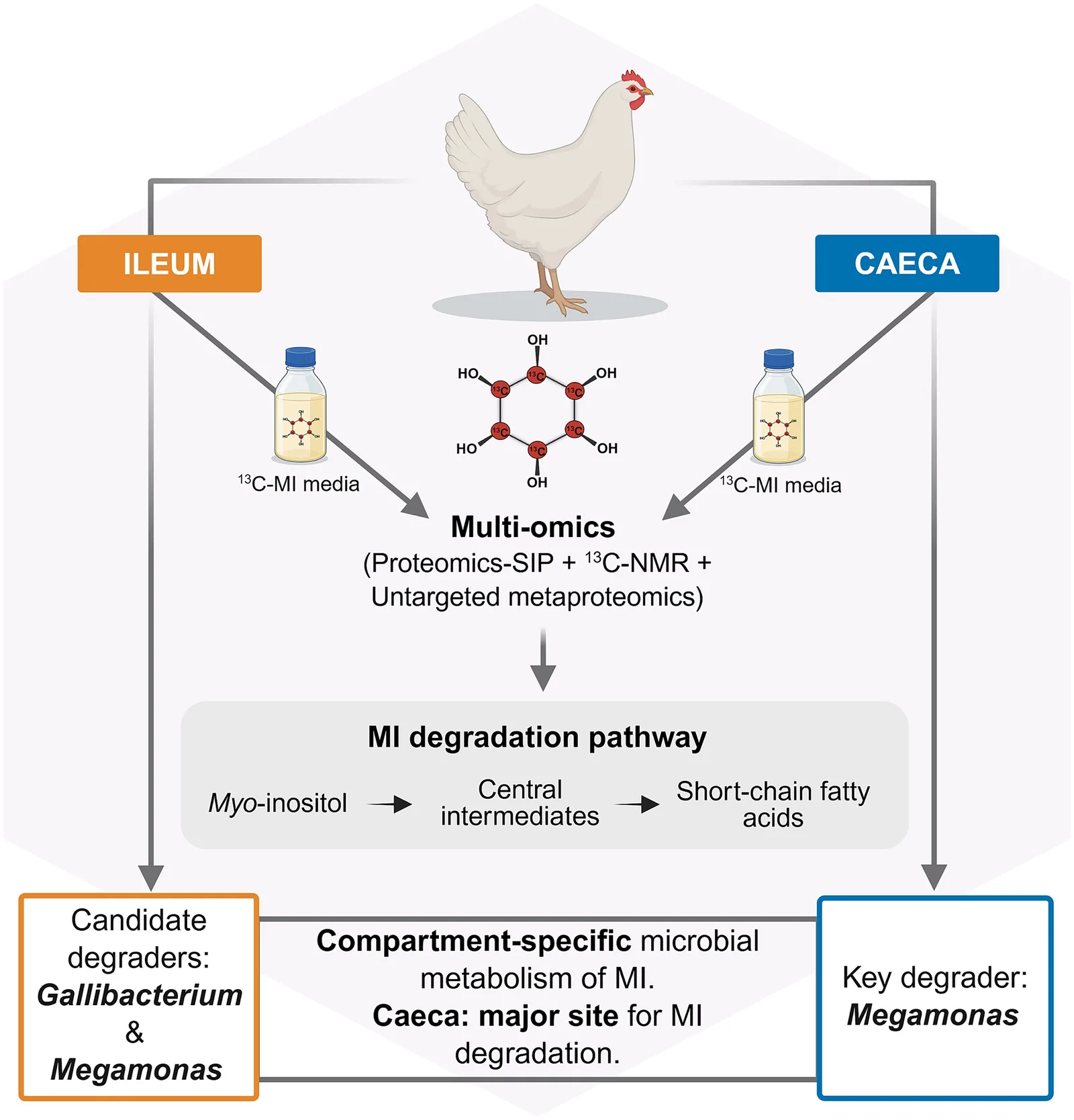

## Introduction

*Myo*-inositol (MI) is an abundant dietary and endogenous carbohydrate that functions as a central metabolite at the interface of host and microbial metabolism. Present both as a free cyclitol and as the headgroup of inositol phosphates (including dietary phytate), MI plays key roles in cellular signalling, osmotic regulation, and membrane lipid structure in animals, and it can constitute a significant carbon pool in the gut following phytate dephosphorylation [1]. Especially in poultry, MI has been reported to have a pivotal role in diverse metabolic, physiological, and regulatory processes, including bone mineralization, breast muscle development, cellular signalling, promotion of ovulation and fertility, modulation of neuronal signalling, and acting as a regulator of glucose homeostasis and insulin regulation [1–3]. Despite its importance, the metabolism of MI in poultry remains poorly understood [4].

Studies have shown that MI serves as a carbon and energy source for microorganisms [5]. To date, mechanistic studies of MI utilization have focused primarily on mammalian gut commensals and plant-associated bacteria. Reviews and experimental reports have documented the pathways used by environmental and gut bacteria to degrade MI, notably the canonical *iol* gene cluster that encodes dehydrogenases, dehydratases, and aldolases converting MI into intermediates which feed into central carbon metabolism and short-chain fatty acid (SCFA) production [6, 7]. Experimental reports have characterized MI degradation in diverse taxa from various environments including *Rhizobium leguminosarum* [8], *Sinorhizobium meliloti* [9], *Lactobacillus casei* [10], *Corynebacterium glutamicum* [11], *Legionella pneumophila* [12], *Citrobacter koseri* [13], *Aerobacter aerogenes* [14], *Bacillus subtilis* [15], *Salmonella enterica* [16], *Anaerostipes rhamnosivorans* [17, 18], *Megamonas rupellensis* [19], *Blautia schinkii* [20], and *Dysosmobacter welbionis* [6]. Large-scale comparative genomics indicated that a substantial fraction of sequenced bacterial genomes carry partial or complete inositol catabolic gene clusters (IolCatGCs), consistent with broad ecological relevance for inositol turnover [21]. However, genomic potential alone does not demonstrate active substrate assimilation in complex microbial communities or reveal which taxa perform specific enzymatic steps under gut conditions.

Isotope-labelling experiments and cultivation studies have shown that intestinal bacteria in mammals can convert MI to acetate, propionate, and other fermentation products, and that these transformations can influence host lipid handling and metabolic endpoints [7, 17, 20]. Evidence for cross-feeding, where primary phytate degraders or phytase-producing strains release MI that is then consumed by other community members, suggests that MI may operate as a shared community resource, shaping trophic interactions in the gut microbiome [5, 18]. Culturomics and co-culture experiments in mammals and livestock underscored this ecological complexity and pointed to candidate taxa that may occupy primary or secondary roles in MI turnover [5, 22]. For poultry, where feed efficiency and nutrient recovery are central to production, understanding how MI is transformed by the gut microbiota is therefore both biologically and practically important.

The chicken gastrointestinal tract is highly compartmentalized, and this spatial structure strongly constrains microbial function. The ileum is characterized by lower biomass and faster transit and typically by communities dominated by *Bacillota* and lactic acid bacteria, whereas the paired caeca are anaerobic fermentation chambers that retain substrates for extended periods and sustain diverse obligate anaerobes and substantial SCFA production in poultry [23]. Consequently, the capacity for MI turnover, the kinetics of substrate consumption, and the identity of active degraders are likely to differ between the ileum and the caeca, with implications for nutrient availability, microbial cross-feeding, and host physiology.

Despite these contextual cues, the identification of MI-assimilating microbes in poultry and the temporal dynamics of MI flux through gut communities remain unresolved experimentally. Metaproteomic stable-isotope probing (SIP) directly links ^13^C-incorporation into newly synthesized proteins with peptide-level identification and taxonomic annotation, thereby assigning substrate assimilation to active taxa and enzymes [24]. Coupling metaproteomic-SIP to ^13^C-metabolite tracing by nuclear magnetic resonance (NMR) provides orthogonal evidence as labelled proteins reveal the microbes and enzymes incorporating MI carbon, while labelled metabolites confirm pathway outputs and flux [25].

We applied an integrated, isotope-resolved multi-omic pipeline to map MI metabolism by ileal and caecal bacterial communities from laying hens to (i) identify taxa that actively assimilate MI carbon, (ii) reconstruct the enzymatic steps of the active taxa, and (iii) evaluate the temporal and spatial partitioning of MI catabolism. Our findings provide the first evidence of active anaerobic MI metabolism, including the spatial partitioning along the chicken gut sections. We established a generalizable workflow to link dietary substrates to microbial function in complex gut ecosystems.

## Materials and methods

### Experimental design

Three Lohmann Selected Leghorn-classic (LSL) laying hens (H1–H3) from the Agricultural Experimental Station of the University of Hohenheim (Unterer Lindenhof, Eningen, Germany) were used in this study. Ileal and caecal digesta were collected aseptically under anaerobic conditions and used to establish microbial enrichment cultures.

Cultures were prepared in a minimal medium containing either no MI, 1 mM unlabelled (^12^C) MI, or 1 mM labelled (^13^C) MI (Supplementary Table S1). Incubations were performed anaerobically at 39°C and monitored over a 36 h time course. Based on preliminary experiments showing limited isotope incorporation in ileal cultures (Supplementary Fig. S1), ileal inocula were subjected to 24 h adaptation step before experimental incubations, whereas caecal cultures were inoculated directly. Fresh digesta collected prior to incubation were analysed by 16S ribosomal RNA gene sequencing to characterize the baseline microbiota. Detailed experimental procedures, medium composition, and analytical workflows are provided in the Supplementary Methods.

### Growth measurement and sample collection

Triplicate cultures were prepared for each hen, intestinal section, medium condition, and sampling time point. Growth was monitored by optical density measurements, and samples collected throughout incubation were separated into supernatant and cell pellets fractions for metabolic and metaproteomic analyses.

### Stable isotope probing and sample analysis

Labelled ^13^C-MI was synthesized from D-[U-^13^C_6_]glucose (99 atom% ^13^C) following Harmel *et al*. [26], and its identity and purity were confirmed by NMR. Incorporation of MI-derived carbon into microbial proteins was determined using a metaproteomic-SIP workflow based on Sipros4 algorithm, while ^13^C-labelled metabolites were analysed by ^13^C-NMR spectroscopy. Complete details regarding sampling, treatments, inoculation, metagenomic data analyses, 16S rRNA gene profiling, MI concentration quantification, metabolomic analysis (NMR), metaproteomic analysis, metaproteomic-SIP data analysis, and metabolic pathway reconstruction of MI degradation are described in the Supplementary Material.

## Results

### Independent survey of ileal and caecal microbiomes in laying hens reveals widespread *myo*-inositol-associated taxa and genes

To establish the ecological and genomic landscape of MI utilization in laying hens, we analysed an independent, untargeted sequencing dataset of ileal and caecal samples from a cohort of 50 Lohmann brown-classic and 50 Lohmann LSL-classic laying hens [27]. Metagenomic assembly and binning recovered metagenome-assembled genomes (MAGs), which were screened for IolCatGCs (Supplementary Fig. S2). Key genes such as *iolG, iolE, iolD*, and *iolB*, were broadly distributed across the cohort and were frequently detected together within the same genomes. By contrast, *iolC* and *iolJ* genes showed more restricted distributions and were not as consistently co-located with the core IolCatGC. Taxonomic assignment of MAGs carrying these genes indicated that *iolG, iolE, iolD*, and *iolB* genes are primarily associated with *Megamonas, Gallibacterium, Staphylococcus*, and *Claveliimonas* lineages. The *iolC* gene was observed principally in *Staphylococcus, Erysipelatoclostridium, Gallibacterium*, and *Claveliimonas* genomes, whereas the *iolJ* gene, encoding the aldolase that links inositol catabolism into central carbon metabolism, was restricted to *Staphylococcus, Limosilactobacillus*, and *Lactobacillus* MAGs. A single taxon, *Staphylococcus agnetis*, was noted to contain the full complement of the IolCatGCs in our survey. The MI transporter gene *iolT* was detected predominantly in *Megamonas* MAGs. Consistent with these gene distributions, species-level abundance patterns differed by intestinal region. *Megamonas* spp. were comparatively more abundant in caecal samples, whereas *Gallibacterium* spp. showed higher relative abundance in the ileum. Overall, taxa encoding IolCatGCs were more frequently observed and more abundant in caecal versus ileal samples in this cohort. Notably, *Megamonas* and *Gallibacterium* emerged as predominant candidate MI degraders in the caeca and ileum, respectively. To verify their potential activities, SIP-based metaproteomic and metabolomic studies were subsequently conducted.

### Baseline microbial community structure of ileal and caecal inocula

The baseline microbial community structure of fresh ileal and caecal digesta samples used as inoculum in the ^13^C-MI SIP incubations was determined, revealing pronounced compositional differences between the sections (Supplementary Fig. S3A). Alpha diversity analysis confirmed that ileal communities were less diverse than caecal communities, with Shannon entropy values of 5–6 and 7–8 (*P* = .0495), respectively, and exhibited greater inter-individual variability among ileal samples compared to caecal samples (Supplementary Fig. S3B). The 10 most abundant genera accounted for ~95% of total relative abundance in ileal samples dominated by *Lactobacillus* (24.3 ± 11%) and related genera, including *Ligilactobacillus* (23.9 ± 18.1%) and *Limosilactobacillus* (13.9 ± 10.5%). In contrast, caecal communities were more taxonomically diverse and included *Lactobacillus* (7.2 ± 4.2%) alongside members of the *Lachnospiraceae* (6.7 ± 0.5%), *Bacteroides* (6.5 ± 1.3%), and *Phocaeicola* (6.4 ± 0.3%).

A greater gene copy number of the bacterial 16S rRNA gene was determined by quantitative polymerase chain reaction (PCR) in all caecal samples, with values ranging from 6.34–6.92 log_10_ copies g^−1^ in the caeca compared to 5.95–6.42 log_10_ copies g^−1^ in the ileum (Supplementary Fig. S3C).

### Growth dynamics of ileal and caecal microbiota and *myo*-inositol consumption under anaerobic conditions

During the *in vitro* incubations, community biomass development was tracked over 36 h via optical density measurements (Fig. 1a). The relatively high initial OD values observed in some caecal cultures likely reflect the intrinsic turbidity of the inoculum, which contained residual feed particles and bacterial aggregates. All treatments were processed using an identical workflow and standardized inoculation conditions. A similar OD pattern was observed in both sections, although there were significant individual differences among the animals regardless of the media used. Ileal-derived enrichment cultures exhibited canonical growth phases (lag, exponential, and stationary) and reached stationary phase by 12 h in all three hens. Caecal-derived enrichment cultures followed a slower, more gradual increase in OD. However, OD reflects bulk culture turbidity and does not directly report MI-specific biomass gain. Importantly, the no-MI control medium still contained peptide-based carbon sources (tryptone-derived components) and therefore represents a carbon-limited but not carbon-free condition.

**Figure 1:**
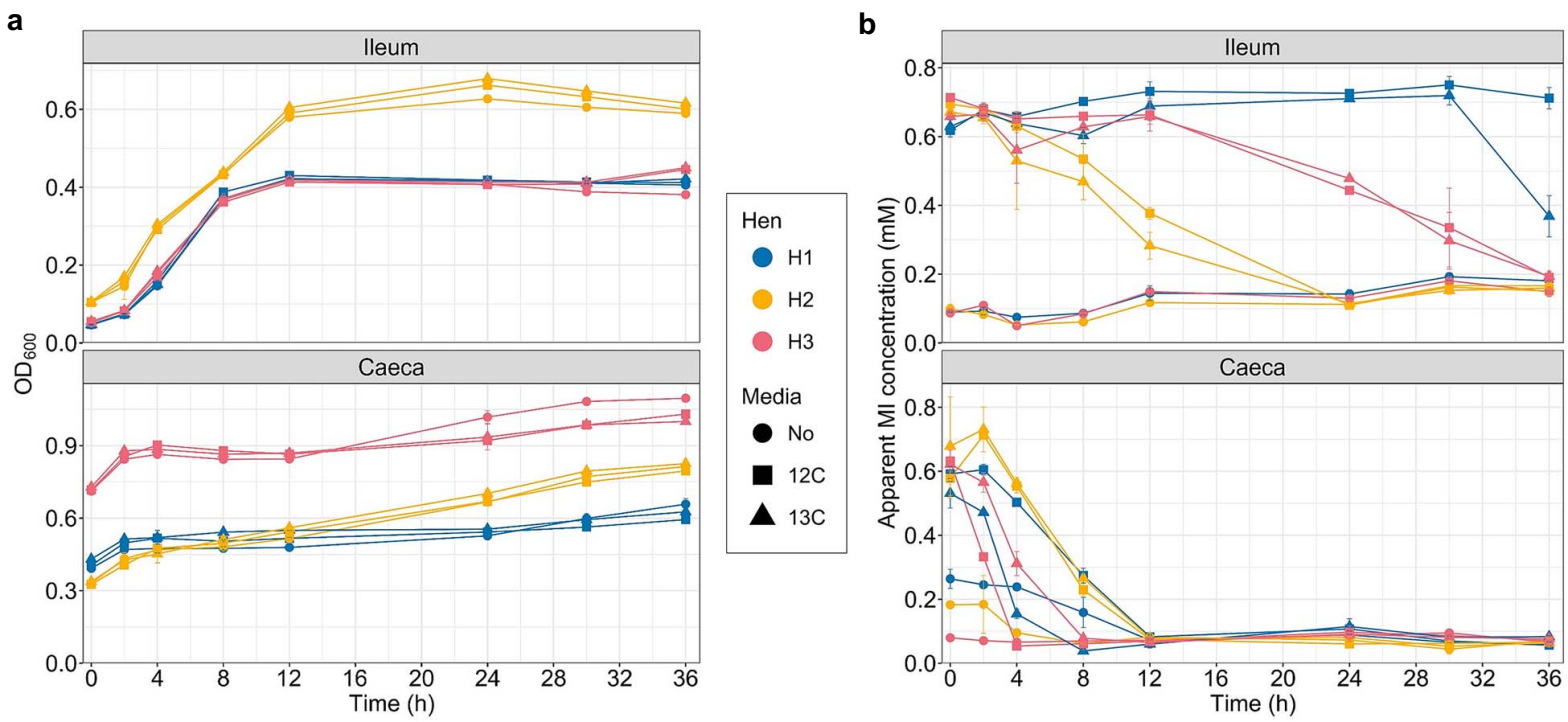
Dynamics of bacterial growth and MI concentration; (a) growth dynamics of ileal- and caecal-derived enrichment cultures; *Y*-axis indicates the optical density at 600 nm (OD_600_) that was measured over 36 h; (b) MI concentration dynamics in culture supernatants over time; *Y*-axis indicates the apparent MI concentration (in mM) that was measured over 36 h; (a and b) *X*-axis indicates the incubation time (in h); data represent mean ± SD of technical replicates (*N* = 3); line colour indicates individual hens, and point shape represents media type; panels show the ileal- (top) and caecal-derived (bottom) enrichment cultures.

MI concentrations in culture supernatants were quantified concomitantly with OD measurements (Fig. 1b). Control medium exhibited a low background signal (~0.11 mM), attributable to matrix effects as expected for cysteine and other reducing components of the medium. This baseline was stable across replicates and substantially lower than MI concentrations observed in experimental treatments. MI concentrations are reported as apparent concentrations (in mM) following unit conversion from a microplate-adapted enzymatic assay that showed a systematic scaling-related underestimation in preliminary validation compared with the standard cuvette format (~40%–45% lower recovery; see Supplementary Methods 1.7). As all samples were processed under identical conditions, the assay reliably captured relative MI depletion kinetics.

MI depletion kinetics differed between sections and between hens. In ileal incubations, MI persisted longest in H1 (detectable at 36 h), whereas near-complete depletion was observed by 24 h in H2 and by 36 h in H3. In caecal incubations, MI was depleted more rapidly, with apparent completion by 12 h in H1 and H2 and by 8 h in H3. The sample specific growth curve and MI depletion curve are presented in Supplementary Fig. S4.

### Comparative ordination of metaproteome and taxonomy-resolved profiles

Metaproteomics analysis of the ileal- and caecal-derived enrichment cultures revealed a total of 38 140 and 35 418 quantified bacterial proteins, respectively (Supplementary Table S2). Principal coordinates analysis based on Bray–Curtis dissimilarities of protein inventories and genome-resolved taxonomic profiles, both derived from metaproteomic data, showed clear condition-dependent structuring of enrichment cultures (Fig. 2 and Supplementary Fig. S5). Permutational multivariate analysis of variance (PERMANOVA; adonis2, 999 permutations) showed statistically significant effects of incubation conditions across all hen and section groups (all *P* = .001). Effect sizes were consistently larger for metaproteome profiles (*R*^2^ range 0.55–0.76) than for taxonomic profiles from metaproteomic data (*R*^2^ range 0.20–0.35), reflecting stronger condition-associated differentiation at the level of expressed protein functions compared to taxonomic composition. Tests for homogeneity of multivariate dispersion (betadisper) were non-significant in all comparisons (*P* > .05), confirming that the observed differences were not driven by unequal within-group variability, indicating that PERMANOVA results reflect centroid shifts rather than differences in within-group dispersion (Supplementary Table S3).

**Figure 2:**
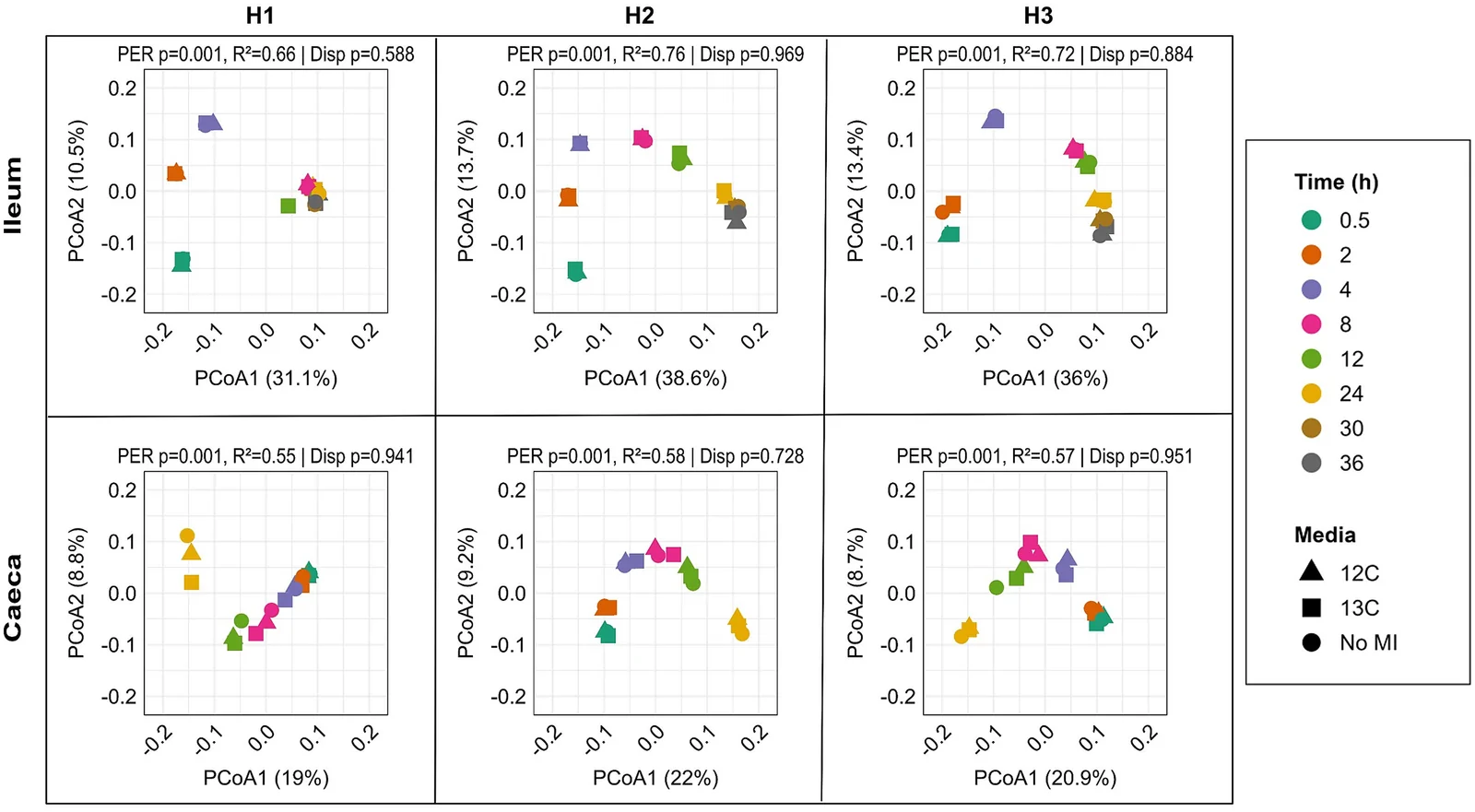
Principal coordinates analysis based on full metaproteome profiles from ileal- and caecal-derived enrichment cultures; each point represents the centroid of triplicate biological samples; shape indicates the medium and colour indicates incubation time (in h); axes represent the first two principal coordinates, with percentage of total variation explained indicated in parentheses; H1, H2, and H3 indicate Hen 1, Hen 2, and Hen 3; PER = PERMANOVA; Disp = dispersion.

Taxonomy profiles reconstructed from the metaproteomic datasets reflected the expected taxonomic characteristics at the section level and revealed additional patterns related to treatment and time (Supplementary Fig. S6A and B). Genera including *Megamonas, Gallibacterium, Ligilactobacillus, Phocaeicola*, and *Bacteroides* exhibited clear compartmentalization between ileal- and caecal-derived enrichment cultures. *Ligilactobacillus* and related taxa were comparatively enriched in ileal-derived cultures, whereas *Megamonas* and *Phocaeicola* were consistently enriched in caecal-derived cultures (Supplementary Fig. S6D and E). A marked exception was *Gallibacterium*, which showed high relative abundance in the ileal-derived cultures from Hen 2 but remained low or undetectable in other individuals and in caecal-derived cultures, highlighting strong inter-individual variability within the ileal microbiota. In caecal enrichment cultures, *Megamonas* relative abundance increased in MI-containing medium during early to mid-incubation and declined at later time points (Supplementary Fig. S7). However, these patterns were observed in the absence of measurable differences in overall biomass development between conditions (Fig. 1a), indicating that responses primarily reflect functional and compositional reorganization within established communities. Together with the similar biomass accumulation observed across media (Section Growth dynamics of ileal and caecal microbiota and MI consumption under anaerobic conditions), these findings indicate that incubation conditions primarily altered microbial metabolic activity rather than driving major changes in community composition. Subsequent SIP-metaproteomic analyses were therefore used to identify the microorganisms actively assimilating MI-derived carbon.

### Detection of ^13^C-labelled proteins across intestinal sections

Proteins incorporating ^13^C-labelled carbon through the assimilation of ^13^C-MI were identified using the SIPROS4 software. The program output was filtered (Supplementary Fig. S8) to obtain a retained dataset using global control removal, as described in the methods section. Across the experiment, 913 and 781 unique labelled proteins were identified in the ileal and caecal datasets, respectively. Clear differences in labelling numbers between intestinal sections and across individual hens were found (Fig. 3a and b). In ileal enrichment cultures, labelled proteins were detected throughout the incubation period, but no consistent temporal trend among hens was observed (Fig. 3a). The timing of peak numbers of labelled proteins varied among individuals: 36 h for H1 (164 proteins), 12 h for H2 (80 proteins), and 30 h for H3 (54 proteins). Although total counts varied among hens, there was a general shift towards higher ^13^C-incorporation levels at later time points, especially of proteins with 10%–70% ^13^C-incorporation. In contrast, the microbial communities in caecal-derived enrichment cultures displayed consistent temporal patterns across hens (Fig. 3b). The number of labelled proteins increased rapidly during early incubation, reached a maximum between 8 and 12 h, and declined by 24 h. Peak labelling occurred for H1 (229 proteins) and H3 (105 proteins) at 8 h, and for H2 (113 proteins) at 12 h. Early time points were dominated by proteins with low ^13^C-incorporation level (5%–6%); however, as incubation progressed, the distribution shifted towards intermediate ^13^C-incorporation levels (8%–40%), while the absolute number of low ^13^C-incorporated proteins remained relatively stable.

**Figure 3:**
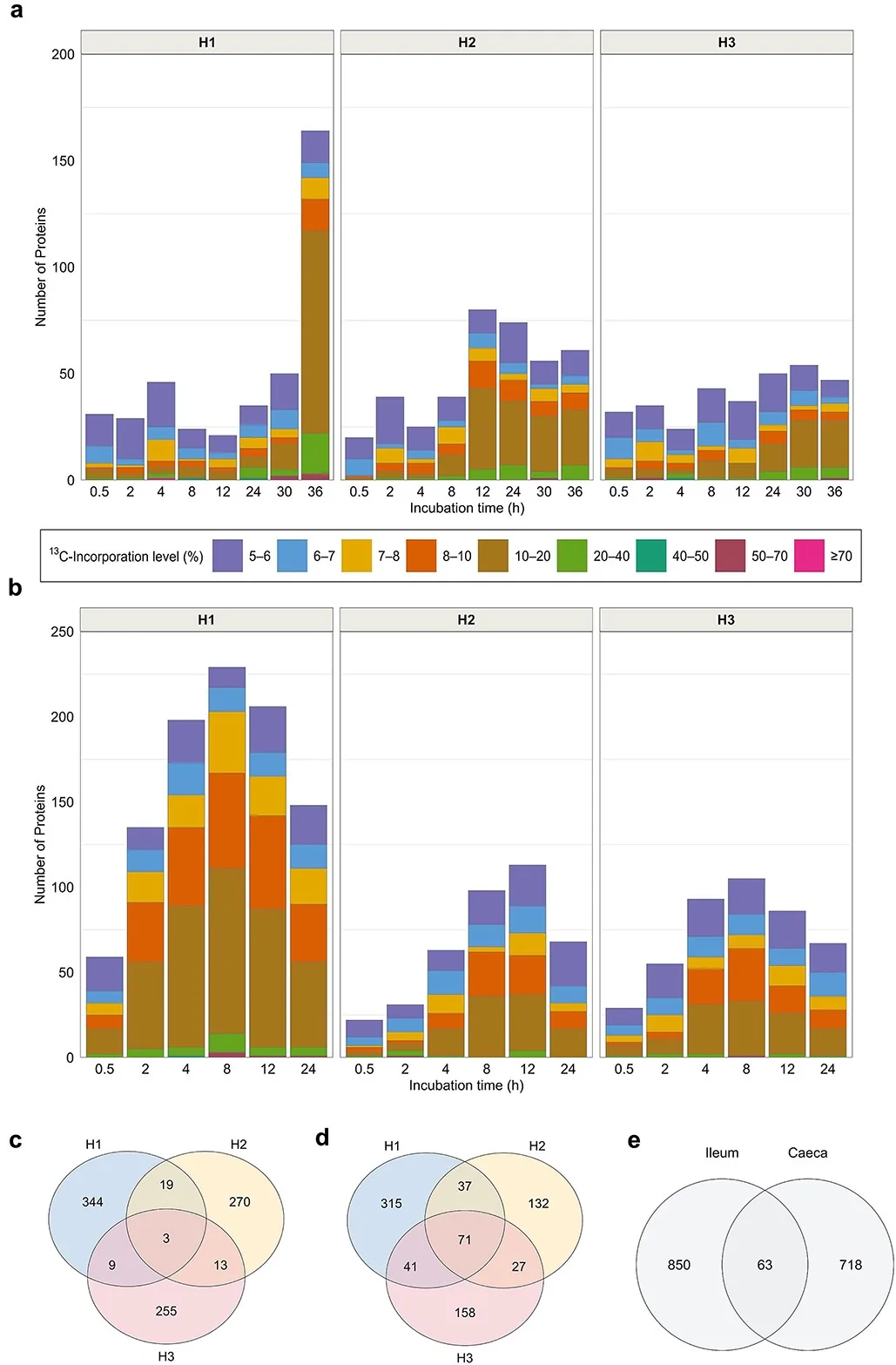
Distribution and overlap of ^13^C-labelled representative proteins across intestinal sections and hens; (a and b) stacked bar plots showing the number of confidently ^13^C-labelled proteins detected per enrichment culture in the (a) ileal-derived and (b) caecal-derived cultures across hens (H1–H3), incubation time points, and ^13^C-labelled media; the *Y*-axis represents the number of representative proteins detected and the *X*-axis represents the incubation time (in h); proteins were grouped according to their average ^13^C-incorporation level (%); protein identification was performed at protein-level FDR thresholds of 0.5% for the unlabelled reference search and 1% for the labelled SIP search; only proteins retained after quality filtering are shown: proteins detected in only one technical replicate were excluded, average ^13^C-incorporation was calculated across technical replicates, proteins with <5% average ^13^C-incorporation were removed, and proteins showing apparent labelling in no-MI or unlabelled-MI control samples were excluded; colours indicate the average ^13^C-incorporation categories (%); (c and d) Venn diagrams showing the overlap of labelled proteins detected across hens within ileal-derived (c) and caecal-derived (d) enrichment cultures after global control removal; (e) Venn diagram showing the overlap of labelled proteins detected between ileal- and caecal-derived cultures after global control removal.

The overlapping analysis revealed marked differences in reproducibility between sections. Ileal datasets exhibited minimal overlaps across hens, with only three proteins shared by all three hens, with 9–19 proteins shared between at least two hens (Fig. 3c). In contrast, caecal datasets showed substantially greater concordance, with 71 proteins common to all hens and 27–41 proteins shared between at least two individuals (Fig. 3d). Comparison between intestinal regions further underscored strong compartmentalization of isotopic labelling, with only 63 labelled proteins common to both sections, while 850 and 718 proteins were exclusively found in ileum and caeca, respectively (Fig. 3e). The overlap analyses group reproducibly labelled proteins within each section and are independent of pathway-level annotation. The distribution of overlapping proteins across hens (unique, shared, and core categories) is provided in Supplementary Table S4.

### Time-resolved taxonomic and functional profile of ^13^C-labelled proteins

The taxonomic origins of ^13^C-labelled proteins in ileal-derived enrichment cultures showed high animal-individual differences among the three hens (Fig. 4a). Each hen exhibited a distinct genus as the major contributor of labelled proteins, H1: *Fusobacterium*, H2: *Gallibacterium*, and H3: *Megamonas*. The distribution of the Clusters of Orthologous Genes (COG) categories among labelled proteins varied accordingly. In contrast, caecal-derived cultures were taxonomically consistent across hens, with *Megamonas* contributing the largest fraction of labelled proteins throughout the incubation (Fig. 4b). The proportion of ^13^C-labelled proteins assigned to *Megamonas* increased during early incubation and declined by 24 h, consistent with the temporal pattern of MI depletion (Fig. 1b).

**Figure 4:**
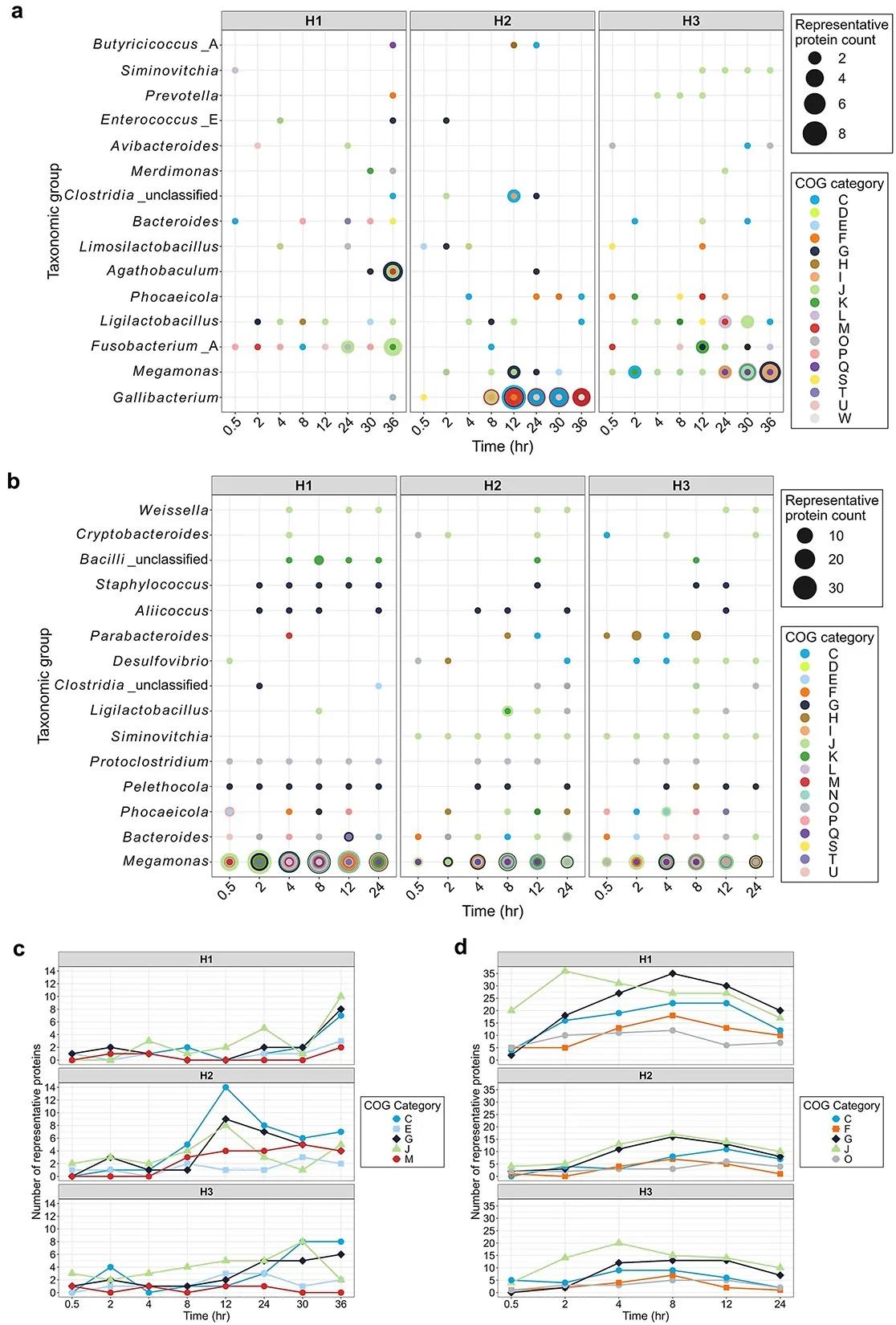
Functional profiles and temporal dynamics of representative labelled proteins in ileal- and caecal-derived enrichment cultures with top taxa and COG dynamics; (a and c) ileum; (b and d) caeca; (a and b) bubble plots showing the distribution of representative labelled proteins across COG categories over time; incubation time is on the *X*-axis and top 15 bacterial taxonomic groups on the *Y*-axis; bubble size reflects the number of representative proteins assigned to each COG category; vertical panels correspond to the three hens; (c and d) temporal trajectories of the five most abundant COG categories (excluding category S) based on representative labelled proteins within each section; *Y*-axis represents the number of representative proteins while *X*-axis represents the incubation time; lines indicate changes in protein counts across incubation time; horizontal panels correspond to the three hens.

Functional classification of labelled proteins showed that COG categories C (energy production and conversion), G (carbohydrate transport and metabolism), and J (translation, ribosomal structure, and biogenesis) were dominant in both intestinal sections (Fig. 4c and d). Notably, COG category G was among the most represented functional groups and encompassed enzymes involved in MI degradation, providing functional context for the observed enrichment. Ileal-derived cultures additionally showed greater representation of COG categories E (amino acid transport and metabolism) and M (cell wall/membrane/envelope biogenesis), whereas caecal-derived cultures were relatively enriched in categories F (nucleotide transport and metabolism) and O (post-translational modification, protein turnover, chaperones), reflecting compartment-specific functional specialization.

Beyond broad functional classification, several ^13^C-labelled proteins were directly associated with the canonical MI degradation pathway. In caecal-derived enrichment cultures, MI catabolism proteins including IolE (K03335), IolD (K03336), and IolB (K03337) were detected and assigned primarily to *Megamonas*, along with IolB to *Pelethocola*. In ileal-derived cultures, IolE and IolD were detected in association with *Gallibacterium* and *Megamonas*, IolB with only *Megamonas*, whereas IolG (K00010) and IolC (K03338)-associated proteins were assigned only to *Gallibacterium*. These findings provide direct evidence that MI-derived carbon was incorporated into proteins belonging to taxa carrying key steps of the Iol pathway. These taxonomic assignments provide functional context for the observed isotope incorporation and are further integrated into the reconstructed MI degradation pathway presented in Section Multi-omics validation of microbial myo-inositol degradation pathways in the hen gut.

Temporal trajectories in labelled protein profiles further demonstrated distinct dynamics between intestinal sections. Ileal-derived cultures showed greater host-specific variability in the timing and composition of labelled proteins, whereas caecal-derived cultures exhibited more reproducible functional trajectories across hens. The top 50 taxa per section and full COG dynamics per hen are provided in Supplementary Fig. S9.

### Metabolomic profiling of ^13^C-*myo*-inositol degradation products

Time-resolved ^13^C-NMR spectroscopy revealed section-specific dynamics of MI turnover and downstream product formation across hens (Fig. 5). Across both intestinal sections, the suite of detected ^13^C-labelled metabolites was largely conserved, except for ^13^C-labelled succinate, which was observed primarily in ileal-derived enrichment cultures (Fig. 5a). Validated reference spectra of detected metabolites are in Supplementary Fig. S10. Across both intestinal sections, depletion of ^13^C-MI over time in media (Fig. 5b) was accompanied by the progressive appearance and accumulation of ^13^C-labelled fermentation products.

**Figure 5:**
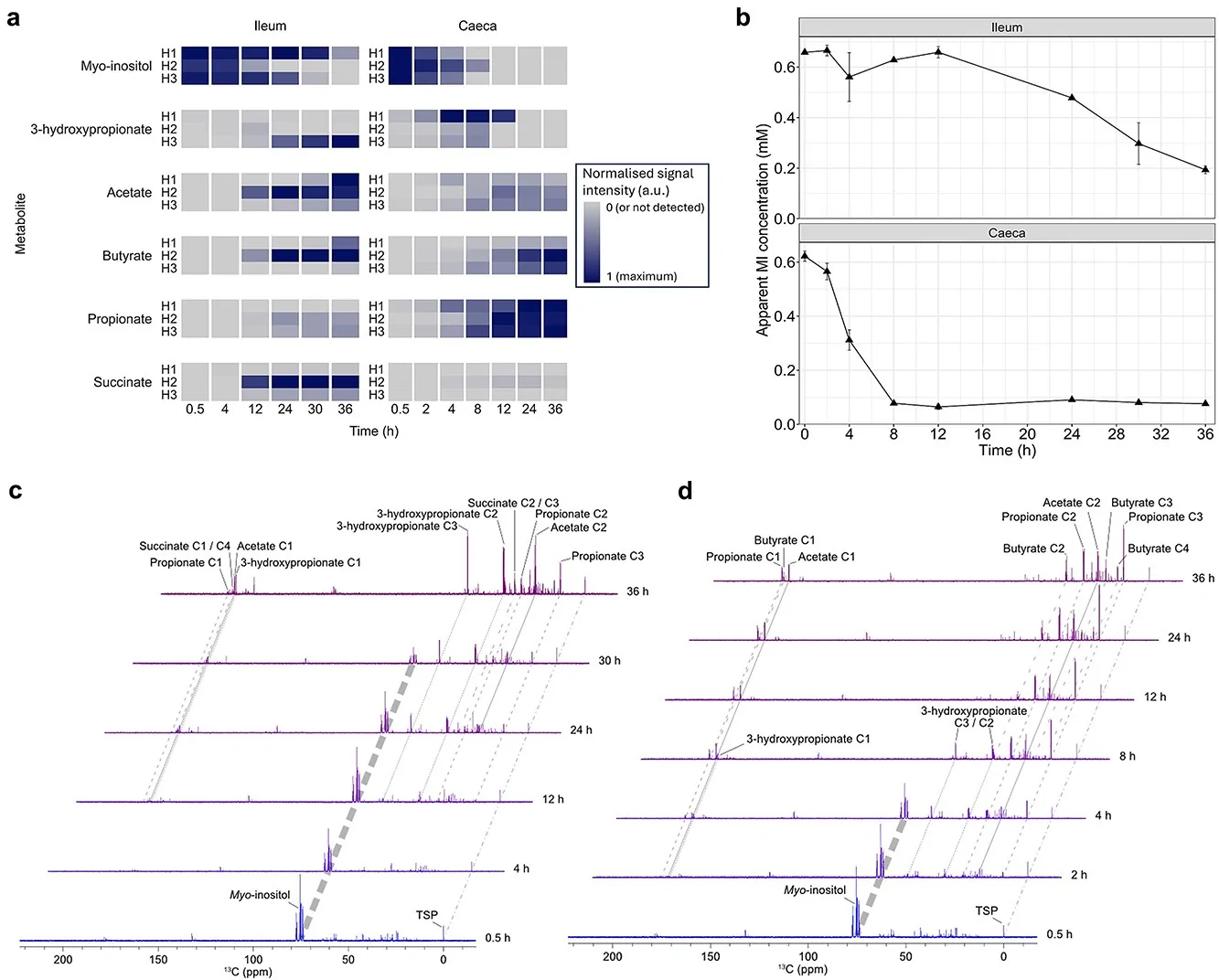
Time-resolved ^13^C-NMR reveals microbial MI catabolism in ileal- and caecal-derived enrichment cultures; (a) semi-quantitative time series of ^13^C-labelled metabolites detected in ileal- and caecal-derived cultures of all hens (H1, H2, and H3); for each metabolite, one or more well-resolved resonance signals were selected for integration; peak integrals were referenced to the internal standard (TSP) within each sample and subsequently normalized for each metabolite to visualize temporal changes in relative abundance; dark blue indicates the highest normalized signal intensity, whereas grey indicates that the metabolite was not detected at the respective time point; because normalization was performed independently for each metabolite, colour intensities represent relative changes within individual metabolites and are not directly comparable between metabolites; (b–d) representative data from H3; (b) subset of Fig. 1b showing time course of apparent MI concentration (in mM) in ^13^C-labelled ileal- and caecal-derived cultures (technical replicates, *N* = 3), shown as mean ± SD; (c and d) overlaid ^13^C-NMR spectra across the time course showing the decline of the ^13^C-MI resonance and the emergence of ^13^C-labelled metabolites in ileal (c) and caecal (d) enrichment cultures; the TSP internal standard remained constant and is included as a chemical-shift reference, confirming consistent spectral calibration across measurements.

In ileal-derived enrichment cultures, MI depletion followed hen-specific trajectories. MI remained detectable through 36 h in H1, whereas near-complete depletion occurred by 24 h in H2 and by 36 h in H3 (Fig. 5a). The first appearance of labelled metabolites was delayed relative to MI consumption, first observed at or after 12 h of incubation. ^13^C-labelled acetate, propionate, butyrate, and succinate were detected, with ^13^C-labelled 3-hydroxypropionate appearing in H3 from 12 h onwards, only weakly detected in H2 at 12 h, and undetected throughout the incubation in H1 (Fig. 5a and c and Supplementary Fig. S11).

In contrast, caecal-derived enrichment cultures exhibited more rapid MI turnover and earlier emergence of labelled metabolites. ^13^C-MI signals disappeared by 8 h in H1 and H3 and by 12 h in H2 (Fig. 5a). In addition to the prominent SCFAs, transient accumulation of ^13^C-labelled 3-hydroxypropionate was observed in several samples, appearing as early as 0.5–2 h but disappearing by 12–24 h (Fig. 5a and d and Supplementary Fig. S11). Labelled butyrate was detected by 24 h in H1 and as early as 4 and 2 h in H2 and H3, respectively. Comparatively, H1 showed the emergence of all metabolites except butyrate and succinate at 0.5 h; however, for other hens, the emergence started by or after 2 h and mostly persisted through the final sampling point.

### Multi-omics validation of microbial *myo*-inositol degradation pathways in the hen gut

Integration of ^13^C-labelled metaproteomic and metabolomic datasets enabled reconstruction of MI degradation pathways in ileal- and caecal-derived enrichment cultures (Fig. 6a and b). The pathway reconstruction was based on confidently identified ^13^C-labelled representative proteins, which were functionally annotated using Kyoto Encyclopedia of Genes and Genomes (KEGG) orthology and linked to their corresponding taxonomic assignments. The detailed pathway, with all reactions and the labelled or unlabelled enzymes present at each reaction step, is provided in Supplementary Fig. S12 and details of the labelled proteins shown in Fig. 6 are provided in Supplementary Table S5.

**Figure 6:**
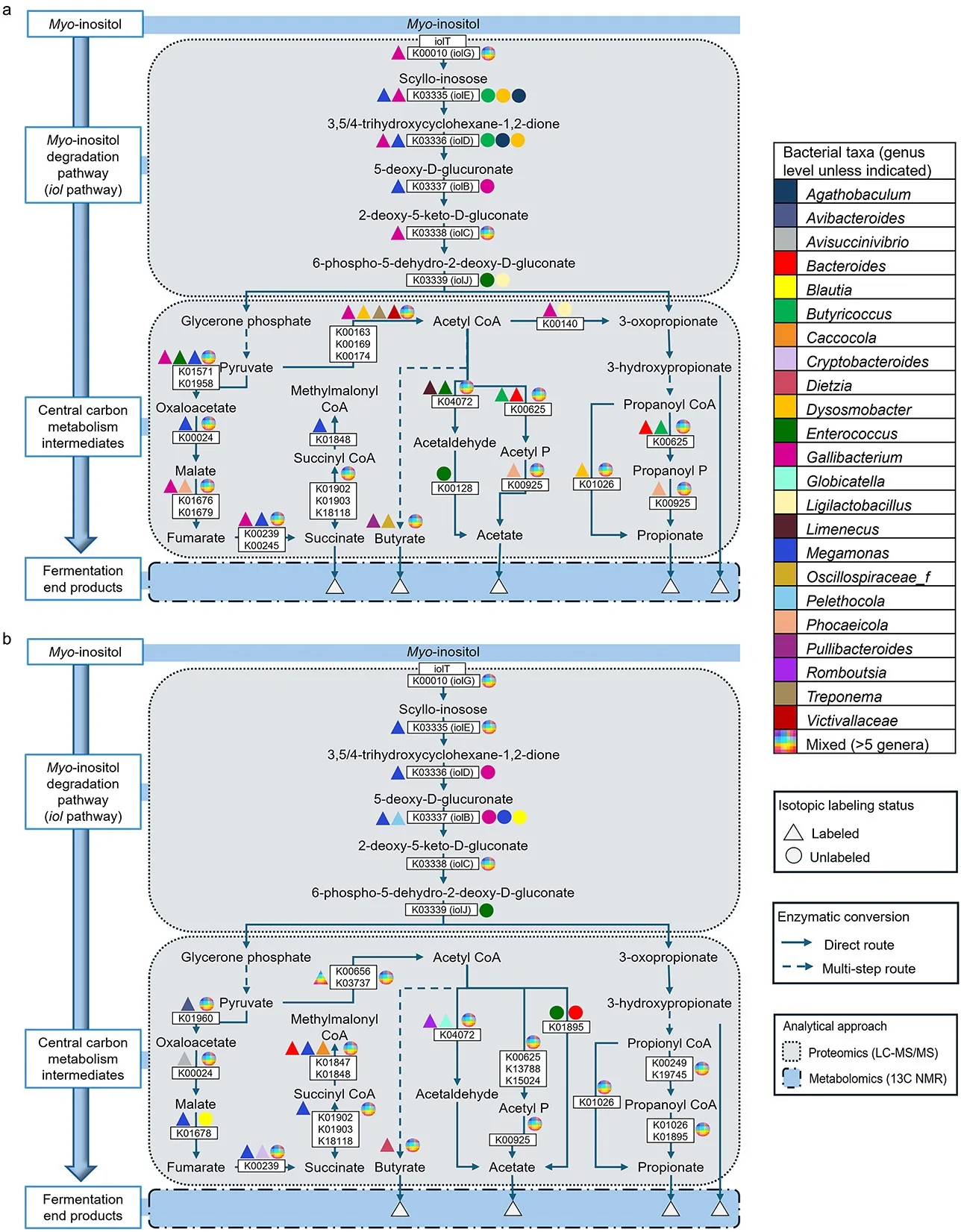
Integrated reconstruction of microbial MI degradation in the hen gut; (a) ileal-derived enrichment cultures; (b) caecal-derived enrichment cultures; pathway diagrams depict the conversion of MI to downstream metabolites through the canonical *iol* pathway and central carbon metabolism; enzymatic steps are based on confidently ^13^C-labelled representative proteins identified by metaproteomic data (MetaLab and SIPROS4) and annotated using KEGG orthology; taxonomic labels are shown at the highest available resolution; entries ending with ‘*_f*’ indicate family-level assignments.

Across both intestinal sections, the core architecture of the canonical MI degradation pathway including the Iol proteins was conserved; however, the taxonomic composition of contributing microorganisms differed. In the ileal-derived enrichment cultures, ^13^C-labelled proteins associated with the *iol* gene pathway were predominantly assigned to *Gallibacterium* and *Megamonas*, whereas in the caecal-derived enrichment cultures, *Megamonas* represented the main contributor, with additional contributions from *Pelethocola* at specific pathway steps. Proteins corresponding to individual pathway reactions were detected in both unlabelled and ^13^C-labelled forms, indicating active incorporation of MI-derived carbon into bacterial proteins. However, the pathway reconstruction represents the functional potential and active carbon assimilation of detected taxa rather than a quantitative measure of pathway flux.

Downstream processing of MI-derived intermediates into central carbon metabolism also exhibited section-specific taxonomic variation. Despite this, a consistent dominance of *Gallibacterium*-associated proteins in the ileal cultures and *Megamonas*-associated proteins in the caecal cultures was observed at multiple steps of carbon flux.

Metabolomic analysis corroborated these findings by confirming the production of ^13^C-labelled acetate, propionate, butyrate, and 3-hydroxypropionate in both intestinal sections, demonstrating active fermentation of MI-derived carbon. In contrast, ^13^C-labelled succinate was detected only in ileal samples. The absence of detectable succinate in caecal samples, despite the presence of identified proteins annotated as K01902, K01903, K18118, K01847, and K01848, suggests rapid metabolic turnover of succinate via conversion to succinyl-Coenzyme A (CoA) and methylmalonyl-CoA.

Together, these results provide convergent evidence from protein-level functional annotation and isotope-resolved metabolite detection, supporting active microbial degradation of MI and its integration into central metabolic pathways in both intestinal sections.

## Discussion

In this study, we investigated microbial MI metabolism using anaerobic enrichment cultures derived from ileal and caecal contents of laying hens and discovered section-specific differences at structural and functional levels. To our knowledge, this is the first combined application of metaproteomic-SIP and ^13^C-NMR to dissect MI turnover in the chicken gut, and the multi-omics integration directly links substrate utilization to active taxa and their metabolic capacities. Metaproteomic-SIP traced ^13^C from MI into newly synthesized proteins, providing taxon-resolved activity evidence [28]. Previous SIP studies have mapped the carbon flow in other host-microbe systems, e.g. glucose and MI in mammalian model animals [6, 29]. However, applying this approach to the chicken gut-derived enrichment cultures revealed a previously unresolved *in situ* pathway of MI catabolism and identified the principal bacterial contributors [1, 4].

The baseline community composition of our samples matched expectations for hens with ileum dominated by *Lactobacillus, Ligilactobacillus, Limosilactobacillus,* and related taxa, and caeca had a richer and more diverse composition, including *Lactobacillus, Limosilactobacillus, Bacteroides, Phocaeicola, Megamonas*, and other taxa, which affirmed the biological relevance of our samples and framed the interpretation of activity data [23, 30–32]. Additionally, individual hen differences were observed in the 16S rRNA amplicon sequencing data from direct samples and later confirmed in metaproteomics and metabolomics results from enrichment cultures. Therefore, we analysed individual hens separately throughout the analysis to account for inter-host variation and avoid overgeneralization.

The data from the present study revealed clear compartmentalization of MI-metabolizing capacity along the gastrointestinal tract in hens. In the caeca, rapid, sustained incorporation of ^13^C from MI into proteins and metabolites, faster depletion of MI, and continuous growth over 36 h were detected, indicating a large and active pool of MI fermenters. In contrast, ileal communities exhibited delayed and often incomplete MI usage, lower early incorporation of the label, and shorter growth windows with a narrower, more specific community composition. Cumulatively, this further explains the presence of a higher number of representative labelled proteins with a broad functional profile in the caecal-derived enrichment cultures than in the ileal-derived enrichment cultures. This finding aligns with the known fermentative capacity of the caeca, with high SCFA production from complex substrates in comparison to the ileum’s simpler and fast-transit community [23]. Furthermore, it confirms and extends the report of MI disappearance to be terminal or post-ileal [4].

Metaproteomic-SIP and temporal labelling consistently identified *Megamonas* as the dominant MI-assimilating genus in caeca across hens. Labelling dynamics (appearance of carbohydrate catabolism enzymes and ribosomal proteins) indicate that *Megamonas* both initiates MI catabolism and supports biomass production in the caecal niche. These functional assignments align with genomic reports that *Megamonas* spp. can carry IolCatGCs [7, 19]. Based on our metaproteome-inferred taxonomy results, the abundance of *Megamonas* increased in MI-medium compared to the control and varied with MI availability (Supplementary Fig. S7). Independent shotgun surveys in a broader cohort metagenomics study corroborated higher prevalence and abundance of taxa containing *iol* genes in caecal compared with ileal samples. In particular, the MAG survey identified iolG, iolE, iolD, and iolB among *Megamonas*-associated genomes from both intestinal regions, consistent with the detection of labelled IolE and IolD proteins in cultures enriched from caecal samples and IolE, IolD, and IolB proteins in cultures from ileal samples. However, some pathway components, including iolC and iolJ, were predominantly associated with other taxa in the MAG dataset and were not detected as *Megamonas*-derived labelled proteins in the SIP experiment. This difference highlights the complementary nature of genome-based predictions and activity-based measurements. MAG analyses indicate metabolic potential within microbial genomes, whereas metaproteomic-SIP captures pathway components actively expressed under the specific enrichment conditions. Variation between datasets may reflect strain-level differences, incomplete pathway reconstruction, differential regulation of pathway genes, or limitations in protein detection.

Ileal activity was more heterogeneous and animal-specific. Metaproteomic-SIP implicated *Gallibacterium* as a candidate MI utilizer in at least one ileal sample, with labelled transporters and stress-response proteins appearing at later time points. Transcriptomic evidence for *iol* clusters in *Gallibacterium anatis* has been reported, indicating that these genes are regulated under acid stress and providing mechanistic plausibility [33]. In addition, *Megamonas,* along with other unexpected taxa, including *Fusobacterium* and *Agathobaculum*, detected in some samples, also carried labelled proteins, suggesting MI degradation in the ileum proceeds via community interactions rather than being dominated by a single, high-abundance degrader. Overall, SIP highlights *Megamonas* as a core caecal MI-utilizer and raises *Gallibacterium* as a candidate ileal MI-assimilator.

Labelled proteins were mainly classified into COGs: energy production (C), carbohydrate metabolism (G), and translation (J), reflecting channelling of MI carbon into central metabolism and growth. Proteins corresponding to canonical MI degradation steps, which are classified within COG category G, were observed to be labelled and were assigned to specific genera, permitting a taxon-resolved pathway reconstruction. Metabolite profiling confirmed expected MI fermentation end-products [6, 7, 18]. This study detected 3-hydroxypropionate in one hen’s ileal sample and its disappearance in caecal time courses, suggesting rapid downstream conversion (e.g. to propionate) [7]. Succinate was observed primarily in ileal incubations and matched the proteomic signal with the absence of labelled enzyme responsible for the conversion of succinate to succinyl-CoA in the ileum but present in caeca, illustrating how combined proteomics and metabolomics resolve pathway bottlenecks and compartment-specific metabolite fate. Previous work documented SCFA formation from MI using limited microbes. Our data extend that knowledge by resolving taxa and temporal steps in a mixed community, while also proving the possible microbial cross-feeding in the complex gut community [6, 7, 18].

In this context, it is important to distinguish between the total metaproteome and the subset of ^13^C-labelled proteins resolved by SIP. While metaproteomic analysis identified a substantially larger pool of expressed proteins across both intestinal sections, only a fraction of these were isotopically labelled and thus directly attributable to MI assimilation. This difference reflects both analytical stringency and biological complexity. From a biological perspective, this disparity is expected in complex gut microbial communities, where only a subset of taxa directly utilize MI as a carbon source, while others may rely on alternative substrates present in the medium, such as peptides derived from tryptone, residual nutrients carried over during sample processing, or cross-feeding [34]. In addition, pre-existing proteins from the inoculum may persist and contribute to the total metaproteome without reflecting active substrate assimilation. Consequently, the labelled proteome provides a functionally resolved view of MI-specific metabolic activity, whereas the total metaproteome reflects the broader and more heterogeneous metabolic landscape of the community.

Integrating metabolomics and metaproteomics allowed multi-layered insight. A summary linking experimental observations to their corresponding interpretations across datasets is provided in Supplementary Table S6. An important observation was the decoupling between community composition and functional activity. Taxonomy inferred from metaproteomic data exhibited comparatively modest changes across media with no significant shifts in community structure. In contrast, protein profiles changed markedly, showing strong time- and substrate-dependent effects. This indicates that microbial function responds more rapidly than composition, consistent with similar observations from protein-based stable isotope approaches [35]. Similar growth dynamics across media further support the interpretation that functional reprogramming, rather than community turnover, drives MI metabolism. Together, this highlights the limitation of composition-based analyses alone and underscores the value of metaproteomic-SIP for capturing rapid metabolic responses.

Findings should be interpreted in the context of several methodological considerations. The sample size was limited, and inter-animal variability was substantial, particularly in the ileum. Accordingly, some taxon-specific interpretations should be considered provisional. The weak MI signal observed in the negative controls in the MI Assay Kit most likely reflects residual carryover from the matrix and assay background in the complex digesta sample and is not attributable to biologically significant MI production. This is consistent with the assay’s known susceptibility to reducing interferents and the greater complexity of the samples, particularly those from the caeca. Furthermore, SIP incubations were performed under *ex vivo* conditions, which required transfer and washing steps that could alter community composition relative to the *in vivo* state. In addition, initial ileal samples were subjected to a 24 h pre-incubation step prior to isotope tracing to increase detectable label incorporation. While this has likely enhanced the activity of MI-responsive populations and may have shifted community structure relative to the original inoculum, direct incubations without pre-adaptation yielded low isotopic incorporation for robust Protein-SIP analysis during preliminary experiments. Importantly, even following pre-incubation, MI utilization in ileal communities remained slower and less extensive than in caecal communities, suggesting that the observed compartment-specific differences reflect underlying biological characteristics rather than the pre-incubation procedure alone. We therefore refrained from overinterpreting baseline 16S rRNA amplicon data in conjunction with SIP-derived correlations and instead emphasized functional patterns that emerged consistently across the multi-omic framework under standardized *in vitro* enrichment conditions rather than as direct measurements of the intestinal lumen *in vivo*.

This study defines, for the first time, that MI metabolism is spatially organized in chickens, with rapid and sustained fermentation in enrichment cultures derived from caecal digesta and delayed, host-specific processing in ileal digesta-derived enrichment cultures. The data confirm the identification of central MI-assimilating genera, *Megamonas* in the caeca and *Gallibacterium* in the ileum, as originally identified in a metagenomic study. It could be demonstrated that metabolic activity can shift independently of community composition. More broadly, this work establishes isotope-resolved, multi-omic pathway reconstruction as a scalable approach to dissect microbial metabolism in complex gut ecosystems. Applying this strategy to other dietary substrates will be essential for resolving how microbiomes transform nutrients and influence host physiology.

## Acknowledgement

We thank Dorothea Fiedler (Leibniz-Forschungsinstitut für Molekulare Pharmakologie) for providing access to facilities and for support with the synthesis of ^13^C-labelled MI, and Meike Amma for assistance during synthesis. We further acknowledge the Mass Spectrometry Group and the NMR Spectroscopy Group at the Core Facility Hohenheim for support with proteomics and NMR measurements, respectively. Additionally, we acknowledge the support of the University of Tübingen and the bwHPC project for computing resources provided by the BinAC cluster. Publishing fees are supported by the Funding Programme Open Access Publishing of the University of Hohenheim.

## Supplementary Material

Supplementary_material_ycag257

## Data Availability

Datasets from the metagenomic survey are available in the European Nucleotide Archive repository (ENA) under the Project ID PRJEB60928. All processed 16S rRNA gene sequences from the six samples have been deposited in the ENA under the Project ID PRJEB107890 (https://www.ebi.ac.uk/ena). Metaproteomics (protein-SIP) data are available in PRIDE (https://www.ebi.ac.uk/pride/); ileum samples: Project accession: PXD077163; caecal samples: Project accession: PXD077115. NMR spectroscopy data are available from the authors upon request.
