# Supplementary material for: Multi-omic stable isotope probing reveals myo-inositol metabolism by anaerobic gut bacteria in laying hens
Source: ISME Commun. 2026 Sep 4;6(1):ycag257. doi: 10.1093/ismeco/ycag257 (PMC13617567; doi:10.1093/ismeco/ycag257)
Supplement: Supplementary file 1 [file supplementary_material_ycag257.zip › Naithani_Supplementary Information_ycag257.pdf]

Multi-omic stable isotope probing reveals *myo*-inositol metabolism by anaerobic gut bacteria in laying hens

Harshita Naithani[1,2], Johannes Günther[3], Timur Yergaliyev[1,2], Amélia Camarinha-Silva[1,2], Jana Seifert[1,2]

[1]Institute of Animal Science, University of Hohenheim, Stuttgart, Germany
[2]HoLMiR - Hohenheim Center for Livestock Microbiome Research, University of Hohenheim, Stuttgart, Germany
[3]Core Facility Spectroscopy, University of Hohenheim, Stuttgart, Germany

1 Materials and methods

1.1 Screening for MI catabolic genes in independent laying hen microbiomes

Metagenome-assembled genomes (MAGs) and their taxonomy and functional annotations (KO), as well as total-per-million (TPM) abundances within GIT sections and hens' breeds, were obtained from another study [1].

1.2 Experimental design and animal procedures

This study was conducted within the interdisciplinary Research Unit P-Fowl: Inositol phosphates and MI in the domestic fowl: Exploring the interface of genetics, physiology, microbiome, and nutrition (project homepage: https://p-fowl.uni-hohenheim.de/en). Three Lohmann LSL-classic (LSL) laying hens housed in group pens at the Agricultural Experimental Station of the University of Hohenheim (Unterer Lindenhof, Eningen, Germany) were used in this study. Birds were maintained under standard husbandry conditions with free access to a commercial layer diet and water. Hens are referred to as H1, H2, and H3 throughout the study. On the day of sampling, hens were anesthetised using a controlled gas mixture (35% CO_2, 35% N_2, and 30% O_2), followed by immediate decapitation, and aseptic excision of ileal and caecal segments was performed immediately thereafter.

To minimise oxygen exposure and prevent introduction of exogenous substrates that could alter microbial activity or bias isotopic labelling, intestinal sections were immediately transferred into separate sterile, nitrogen-flushed flasks containing MI-free minimal growth medium with 0.001 gL^{-1} resazurin as a redox indicator (medium formulation in Supplementary Table S1 adapted from Naithani et al. [2]). During transportation, samples were maintained under anaerobic conditions at 35-40°C and processed immediately upon arrival in the laboratory in accordance to our previous work [3].

1.3 Growth media

Three minimal growth media were prepared based on the formulation of minimal broth medium described by Naithani et al. [2], with modifications restricted to the presence, concentration, and isotopic composition of MI, which served as the principal added carbon source (Supplementary Table S1) [2]. The first medium lacked MI and served as both a negative control and the transport medium. The second medium contained 1 mM unlabelled (^{12}C) MI, and the third contained 1 mM labelled (^{13}C) MI. MI concentration of 1 mM was used as a moderate tracer-compatible substrate input to enable isotope detection without imposing strong enrichment bias. The ^{13}C-labelled MI was synthesised from D-glucose (U-$^{13}C_6$, 99 atom% ^{13}C; Cambridge Isotope Laboratories Inc.,) following Harmel et al. (2019) at Leibniz-Forschungsinstitut für Molekulare Pharmakologie, Germany [4]. The product identity and chemical purity were verified by NMR prior to use, confirming structural identity and absence of detectable impurities.

All media were sterilised by autoclaving at 121°C for 15 min at 15 psi, then sparged with nitrogen to establish anaerobic conditions.

1.4 Sample preparation, treatment and inoculation

All subsequent procedures were performed inside an anaerobic chamber (Whitley A35 workstation, Don Whitley Scientific, UK) containing a mixture of 80% N_2 (quality level 5.0), 15% CO_2 (quality level 3.0) and 5% H_2 (quality level 5.0). The sections were cut open using sterile scissors, and the entire ileal and caecal digesta fraction from each bird were collected by gently scraping the intestinal content using sterile spatulas under aseptic conditions. A subsample of fresh digesta was transferred directly into a sterile tube for 16S rRNA analysis and immediately stored at -80°C. The remaining digesta were suspended in the corresponding MI-free transport medium, gently agitated for 5 min in a shaker, and then filtered through sterile four-layer cheesecloth to remove large particles. The residue was washed with minimal growth medium containing unlabelled MI. The resulting filtrate served as the microbial inoculum.

Two equal sets of Hungate tubes per medium were prepared for the subsequent incubations, one for optical density measurements and the other for MI quantification, metaproteomics, and metabolomic analyses. For all experiments, three Hungate tubes per time point, gut section, medium, and hen were used and considered as technical replicates for this study. All tubes were incubated at 39°C under anaerobic conditions for different time periods.

For the caecal samples, the freshly acquired inoculum was directly transferred into separate flasks at 2% (v/v) containing each of the three media. Following homogenisation, inoculated medium from each flask were dispensed into Hungate tubes at equal volume (10 mL) under anaerobic conditions.

Ileal samples followed the same workflow with one modification. Preliminary experiments demonstrated reduced ^{13}C incorporation into microbial proteins in ileal samples without prior adaptation, whereas caecal samples exhibited robust isotopic incorporation irrespective of pre-incubation (Supplementary Fig. S1). Therefore, after filtration and washing of residue with unlabelled MI media, ileal filtrate was incubated anaerobically for 24 h to enhance detectable isotopic incorporation. The pre-incubated filtrate was then used as inoculum for further experimental incubations, as described for caecal samples.

1.5 Growth measurement and sample collection

Triplicate Hungate tubes were prepared for each medium type and gut section from each hen. Incubations were carried out at 39°C, and optical density at 600 nm (OD_{600}) was measured at 0, 2, 4, 8, 12, 24, 30, and 36 h in the same set of Hungate tubes to prevent any variation. Prior to each measurement, tubes were thoroughly mixed to ensure homogeneous suspension. Growth curves were generated from these sequential measurements using R (version 4.3.1) using the mean of the technical replicates, and error bars to represent ± standard deviation (SD).

At 0.5, 2, 4, 8, 12, 24, 30, and 36 h of incubation, three Hungate tubes per hen, intestinal section, and medium were taken for downstream analyses. Cultures were centrifuged at 2500 g for 15 min at 4°C. Supernatants were transferred into sterile tubes and used for subsequent MI quantification and metabolomic analyses. Cell pellets were reserved for metaproteomic and protein-SIP analysis. Both fractions were stored at -80°C until further processing. All other procedures including metagenomic data analyses, 16S rRNA gene profiling, MI concentration quantification, metabolomic analysis (NMR), metaproteomic analysis, metaproteomic-SIP data analysis and metabolic pathway reconstruction of MI degradation are described in the Supplementary Material.

1.6 16S rRNA gene profiling

DNA extraction and 16S rRNA gene sequencing and qPCR-based bacterial abundance estimation

For analysis of initial microbial communities in the sample, the DNA extraction of the six digesta samples (three hen, two sections) was performed using QIAamp PowerFecal Pro DNA kit (Qiagen, Germany) following the manufacturer's instructions. The extracted DNA was quantified using NanoDrop One (Thermo Fisher Scientific, Wilmington, DE) and used for both 16S rRNA gene sequencing and quantitative PCR (qPCR)-based estimation of bacterial 16S rRNA gene abundance.

Bacterial community composition was assessed by sequencing the V1–V2 hypervariable region of the 16S rRNA gene. Amplicon generation and library preparation were performed using a two-step PCR protocol as previously described by Kaewtapee et al [5]. Briefly, the

target region was amplified using region-specific primers, followed by a second PCR step to incorporate sequencing adapters and sample-specific indices. Further, amplicon size and integrity were verified by agarose gel electrophoresis. PCR products were normalised using the SequalPrep Normalization Plate Kit (Invitrogen, Carlsbad, CA, USA) and subsequently purified with the MinElute PCR Purification Kit (Qiagen, Hilden, Germany). Libraries were sequenced on an Illumina NovaSeq platform (Illumina Inc., San Diego, CA, USA) using 250 bp paired-end reads.

Bacterial 16S rRNA gene copy numbers were quantified by qPCR using universal bacterial primers U2-F (5′-ACTCCTACGGGAGGCAG-3′) and U2-R (5′-GACTACCAGGGTATCTAATCC-3′), generating an approximately 450 bp amplicon [6]. qPCR was performed using PowerTrack SYBR Green Master Mix (Thermo Fisher Scientific). The amplification program consisted of an initial denaturation at 95°C for 5 min, followed by 30 cycles of denaturation at 95°C for 20 s and annealing at 56°C for 1 min. DNA samples were analysed at a 1:100 dilution. Amplification data were processed and bacterial 16S rRNA gene abundance was expressed as copies per gram of sample as described by Neves et al. (2025) [6].

16S rRNA data analysis

Raw reads were demultiplexed with Sabre (https://github.com/najoshi/sabre) and then imported into Qiime2 [7]. Primer sequences were removed using q2-cutadapt [8]. Sequence denoising, pair-end merging, and chimeras' removal were performed using q2-DADA2, generating high-resolution amplicon sequence variants (ASVs) [9]. Taxonomic assignments of ASVs was conducted using the Bayesian classifier trained on the GTDB (v. 226) [10].

1.7 MI concentration quantification

MI concentrations were determined using a commercially available enzymatic MI assay kit (Megazyme Ltd., Wicklow, Ireland), according to the manufacturer's instructions. The assay is based on an INT-coupled enzymatic reaction measured spectrophotometrically at 492 nm. All samples were within the 2-35 µg MI range (equivalent to 0.02-0.4 g/L in the standard format), which the manufacturer recommended as the linear range.

The assay was downscaled to one-tenth of the recommended reaction volume for microplate-based measurement, following previously described adaptations by Röhm et al. (2022) [11]. Absorbance was recorded at 492 nm, and MI concentrations were calculated using the manufacturer's equations.

Preliminary validation using an MI standard (0.25 g/L) showed that the downscaled microplate format yielded a measured value of 0.14 g/L, whereas the standard cuvette-based assay recovered the expected concentration, indicating a systematic scaling-related underestimation. To verify the starting substrate concentration, the initial culture medium was

additionally analysed using the standard cuvette assay and by NMR, both confirming an initial MI concentration of 1 ± 0.2 mM. Accordingly, the microplate assay was used to assess relative MI depletion kinetics rather than absolute concentrations under identical conditions.

In addition, due to the complexity of digesta-derived matrices, residual reducing and low-molecular-weight compounds may contribute to background signal. Therefore, all measurements were interpreted relative to matrix-matched controls.

Assay precision was assessed empirically through repeated measurements of selected samples. Intra-assay and inter-assay coefficients of variation were 5.22% and 4.55%, respectively.

1.8 Metabolomic analysis

NMR sample preparation and processing

Time points analysed by ^{13}C NMR were selected based on MI depletion kinetics. For caecal cultures, samples from 0.5, 2, 4, 8, 12, 24, and 36 h were examined. For ileal cultures, samples from 0.5, 4, 12, 24, 30, and 36 h were analysed.

10 mL of each sample was centrifuged at 10,000 g for 30 min at 4°C to remove residual particulate material. The supernatant was filtered through a 0.2 µm cellulose acetate syringe filter to eliminate remaining cells. Filtered supernatants were aliquoted and dried under vacuum at room temperature using a SpeedVac concentrator. On the day of analysis, dried extracts were reconstituted in 750 µL deuterated phosphate buffer prepared in D_2O. Samples were vortexed vigorously until fully dissolved. After centrifugation at 13,000 g for 30 min at 4 °C, 500 µL of the clarified supernatant was transferred into 5 mm NMR tubes and stored at 4 °C until measurement. The phosphate buffer used was prepared in D_2O using KH_2PO_4 and K_2HPO_4 to achieve the desired pH. Trimethylsilyl propionate (TSP) was included as an internal chemical shift reference.

Carbon NMR spectroscopy and data analysis

NMR measurements were recorded on a Bruker 600 MHz spectrometer (Avance III HD; Bruker Biospin), equipped with a 5 mm Prodigy BBO cryoprobe at a ^{13}C frequency of 151 MHz at the Core Facility Hohenheim. The one-dimensional ^{13}C experiments were recorded with a Bruker pulse program with heteronuclear decoupling. Spectra were recorded at 298 K with 512 scans and a spectral width from -20 ppm to 220 ppm.

Data acquisition and processing were performed using TopSpin 3.7.0 and 4.5.0 (Bruker Biospin, Germany), respectively. Processing involved automatic phase and baseline correction. Metabolite assignments were initially based on comparison of observed ^{13}C chemical shifts with published values and publicly available spectral databases (https://bmrb.io). Structural identities were further supported by acquiring 1D and heteronuclear 2D HSQC NMR spectra of authentic, unlabelled reference compounds under

identical buffer (D_2O phosphate buffer) and acquisition conditions and comparing chemical shift positions. The reference compounds used to validate NMR assignments were sodium acetate trihydrate (Carl Roth GmbH), succinic acid (Sigma-Aldrich Chemie GmbH), butyric acid (Sigma-Aldrich Chemie GmbH), propionic acid (Carl Roth GmbH), and 3-hydroxypropionic acid (Fluorochem) for acetate, succinate, butyrate, propionate and 3-hydroxypropionate, respectively. Processed spectra were imported to Affinity Designer (v. 1.10; Serif Ltd.) to add metabolite trace lines and for figure preparation.

1.9 Metaproteomic analysis

Protein extraction and purification

For downstream analyses, bacterial cultures (10 mL per condition) were harvested at time points based on MI depletion kinetics in the corresponding supernatants. Bacterial pellets from caecal cultures were processed from 0.5, 2, 4, 8, 12, and 24 h. For ileal cultures, samples from 0.5, 2, 4, 8, 12, 24, 30, and 36 h were included to capture the extended MI depletion phase observed in this section.

Pellets were washed with phosphate-buffered saline (PBS) to remove residual medium components. Briefly, pellets were resuspended 1 mL PBS, vortexed, and centrifuged at 12,500 g for 30 min at room temperature. The supernatant was discarded and pellets were resuspended in 200 µL of extraction buffer (20 mM Tris-HCl, 2% SDS, pH 7.5). Protein extraction and quantification were performed following the protocol described by Saenz et al (2021) [12]. Protein purification was carried out by SDS-PAGE followed by in-gel digestion. Peptides were further cleaned using five-layer C18 layer stage-tip purification, eluted, dried in a SpeedVac, and stored at -20°C until mass spectrometry analysis.

Liquid chromatography with tandem mass spectrometry (LC-MS/MS) analysis

Peptide samples were analysed on an Ultimate 3000 nano-RSLC system (Thermo Fisher Scientific, Sindelfingen, Germany) coupled to an Orbitrap Exploris 480 mass spectrometer (Thermo Fisher Scientific, Sindelfingen, Germany) using a Nanospray-Flex ion source (Thermo Fisher Scientific, Sindelfingen, Germany). Solvent A was 0.1% formic acid in water, and solvent B was 80% acetonitrile with 0.1% formic acid. Chromatographic separation was performed at a flow rate of 0.30 µL min^{-1} and a column temperature of 35 °C. The 60 min gradient was programmed as follows: 0-2 min, 2% B; 2-16 min, 2-21% B; 16-35 min, 21-36% B; 35-44 min, 36-50% B; 44-48 min, 50-96% B; 48-50 min, 96% B; 50-53 min, 96-2% B; 53-60 min, 2% B. Sample injection volumes were adjusted individually to load approximately 250 ng total peptide per run. The Exploris 480 acquired MS1 scans over an m/z range of 300 to 1600 at 120,000 resolutions (AGC target 1,000,000; maximum injection time 50 ms). Precursors with intensities ≥300,000 and charge states 2 to 4 were selected for data-dependent acquisition, with a 30 s dynamic exclusion and up to 25 MS/MS scans per cycle. Fragmentation

was performed using higher-energy collisional dissociation at 30% normalised collision energy. MS/MS spectra were acquired with a 1.6 m/z isolation window at 15,000 resolution (AGC target 90,000; maximum injection time 40 ms).

Metaproteomic data analysis

Peptide and protein identification, quantification, taxonomic assignment, and functional annotation were performed using MetaLab-MAG v.1.0.2 [13]. Microbial protein identification was performed using a publicly available chicken gut microbiome database (v1.0.1) from Mgnify [14], and sample-specific protein databases were automatically generated by MetaLab [13] through its spectral clustering algorithm to optimise peptide assignment. Host-derived proteins were identified against the *Gallus gallus* reference proteome (UniProt: UP000000539; 18,372 protein-coding genes). Label-free quantification (LFQ) values were obtained using the default settings in MetaLab. Peptides and proteins were filtered at a 1% false discovery rate (FDR), and protein-level quantification was obtained using the MaxLFQ algorithm [15] integrated in MetaLab. Carbamidomethylation of cysteine residues was applied as a fixed modification, while methionine oxidation and N-terminal acetylation were included as variable modifications.

To calculate the total number of representative bacterial proteins per section in the ^{13}C-MI media, protein IDs lacking the 'MGYG' prefix were removed, and distinct protein IDs per section were counted using the distinct() function from the dplyr package in R. This yielded the total number of bacterial proteins detected, irrespective of isoforms or redundant identifications.

For comparative analyses, technical replicates were summarised to a single biological measurement by taking the median protein intensity per biological sample. Protein intensities were transformed as log10(intensity + 1) prior to multivariate analysis to stabilise variance and reduce the influence of highly abundant proteins.

Two complementary abundance matrices were analysed: (i) a protein-level matrix representing functional profiles and (ii) a genome-resolved taxonomy matrix derived from MetaLab, representing taxonomic composition. For both datasets, Bray-Curtis dissimilarities [16] were calculated on the transformed abundance values to quantify differences in community structure using the vegan R package (v2.6.8) [17].

To assess the effects of incubation conditions, permutational multivariate analysis of variance (PERMANOVA) [18] was performed using the adonis2 function in the vegan R package (v2.6.8) [17] with 999 permutations separately for each hen and intestinal section, with media, time, and their interaction included as explanatory variables. PERMANOVA models were fitted separately for each hen–section subset. Homogeneity of multivariate dispersion was evaluated using the PERMDISP method [19] via the betadisper function [17] with permutation testing to confirm that observed differences were not driven by unequal within-group variability. For each model, R^2 values and permutation-based P values were reported.

Metaproteomic-SIP data analysis

Isotopic incorporation of ^{13}C into microbial proteins was quantified using SIPROS4, following the workflow described by Xiong et al. (2024) [20], with modifications implemented in the present study. The customised workflow is available at https://github.com/Harshita-Naithani/SIP_Laying-hen.git. For each hen, gut section, and time point, the corresponding unlabelled sample was used as the reference input for creating a sample-specific database. Protein identification was performed against a chicken gut protein database obtained from MGnify (chicken gut v1-0-1, protein catalogue 95) [14], supplemented with common laboratory contaminants from the cRAP database [21]. SIPROS4-generated pro2psm.cluster files were used to quantify peptide-level isotopic patterns and protein-resolved average ^{13}C-incorporation levels. Quality filtering was applied to restrict the analysis to confidently labelled proteins. Protein identification and quantification were performed using SIPROS4 default filtering procedures, including peptide-spectrum match (PSM) filtering and protein-level false discovery rate (FDR) control. Protein-level FDR thresholds of 0.5% for the unlabelled reference search and 1% for the labelled SIP search were applied during SIPROS4 processing. Proteins detected in only one technical replicate were removed to ensure reproducible detection. Within-sample redundancy was collapsed by averaging ^{13}C-incorporation level for duplicate identifiers, followed by averaging across technical replicates.

A minimum average ^{13}C-incorporation threshold of 5% was applied to define labelled proteins This threshold was selected based on the expected natural abundance of ^{13}C (~1.1%) and the low-level variation observed in control incubations lacking labelled MI. Additionally, protein showing apparent labelling in control samples lacking MI or containing unlabelled MI was also discarded from the corresponding intestinal section datasets. Therefore, only proteins displaying reproducible isotope incorporation above background levels in ^{13}C-MI incubations were retained for downstream analysis. Reverse MGnify entries (prefixed “Rev_”) were removed during the filtering steps.

Functional annotation of labelled proteins was performed by mapping MGYG identifiers to MGnify-provided metadata tables [22], which include EggNOG orthology assignments [23], COG categories [24], KEGG [25] and EC entries [26], and Pfam domains [27]. COG functional categories were assigned according to the NCBI COG database [24]. The resulting annotated protein dataset was used for all downstream taxonomic and functional analyses.

1.10 Metabolic pathway reconstruction of MI degradation

Representative ^{13}C-labelled proteins retained after the SIPROS4 quality-filtering workflow were used for metabolic pathway reconstruction. Functional annotation was performed by assigning KEGG orthology (KO) [25] identifiers through integration of EggNOG annotations [23] with the MGnify chicken gut genome data [14] to assign taxonomic information. The resulting KO list

was submitted to iPath3 [28] to visualise the metabolic network and identify enzymatic reactions supported by the labelled proteins and determine pathway steps lacking direct ^{13}C-labelled protein evidence.

To further contextualise pathway completeness, the corresponding enzymes required for the MI degradation pathway including both detected and missing labelled proteins, were searched within the unlabelled metaproteomic dataset. Taxa contributing to at least two unlabelled proteins associated with the reconstructed pathway were retained for visualization. The final pathway figure was manually curated by mapping detected enzymatic steps onto the MI degradation pathway and annotating each reaction with the corresponding bacterial genera inferred from protein-level taxonomic assignments. The fermentation end products were included from metabolomic dataset. The resulting reconstruction represents a qualitative overview of MI degradation potential supported by metaproteomic-SIP evidence and associated taxonomic context, rather than a quantitative representation of protein abundance or pathway flux.

References

1. Yergalyiev T, Roth C, Rodehutscord M, Seifert J, Camarinha-Silva A. Age, strain, and gut section shape the microbiome of commercial laying hens. *Poult Sci* 2026;**105**:107152. https://doi.org/10.1016/j.psj.2026.107152
2. Naithani H, Rios-Galicia B, Silva AC, Seifert J. Strategies to enhance cultivation of anaerobic bacteria from gastrointestinal tract of chicken. *J Vis Exp JoVE* 2024;e66570. https://doi.org/10.3791/66570
3. Naithani H, Camarinha-Silva A, Fuchs TM, Seifert J. Isolation and molecular identification of potential *myo*-inositol-utilizing bacteria from the gastrointestinal tract of laying hens. *Microbiol Res* 2026;**17**:46. https://doi.org/10.3390/microbiolres17030046
4. K. Harmel R, Puschmann R, Trung MN, Saiardi A, Schmieder P, Fiedler D. Harnessing ^{13}C-labeled *myo*-inositol to interrogate inositol phosphate messengers by NMR. *Chem Sci* 2019;**10**:5267-74. https://doi.org/10.1039/C9SC00151D
5. Kaewtapee C, Burbach K, Tomforde G, Hartinger T, Camarinha-Silva A, Heinritz S, et al. Effect of *Bacillus subtilis* and *Bacillus licheniformis* supplementation in diets with low- and high-protein content on ileal crude protein and amino acid digestibility and intestinal microbiota composition of growing pigs. *J Anim Sci Biotechnol* 2017;**8**:37. https://doi.org/10.1186/s40104-017-0168-2
6. Neves ALA, Vieira RAM, Vargas-Bello-Pérez E, Chen Y, McAllister T, Ominski KH, et al. Impact of feed composition on rumen microbial dynamics and phenotypic traits in beef cattle. *Microorganisms* 2025;**13**:310. https://doi.org/10.3390/microorganisms13020310

7. Bolyen E, Rideout JR, Dillon MR, Bokulich NA, Abnet CC, Al-Ghalith GA, et al. Reproducible, interactive, scalable and extensible microbiome data science using QIIME 2. *Nat Biotechnol* 2019;**37**:852-57. https://doi.org/10.1038/s41587-019-0209-9
8. Martin M. Cutadapt removes adapter sequences from high-throughput sequencing reads. *EMBnet.journal* 2011;**17**:10-12. https://doi.org/10.14806/ej.17.1.200
9. Callahan BJ, McMurdie PJ, Rosen MJ, Han AW, Johnson AJA, Holmes SP. DADA2: High-resolution sample inference from Illumina amplicon data. *Nat Methods* 2016;**13**:581-83. https://doi.org/10.1038/nmeth.3869
10. Parks DH, Chuvochina M, Chaumeil P-A, Rinke C, Mussig AJ, Hugenholtz P. A complete domain-to-species taxonomy for bacteria and archaea. *Nat Biotechnol* 2020;**38**:1079-86. https://doi.org/10.1038/s41587-020-0501-8
11. Röhm K, Gonzalez-Uarquin F, Harmel RK, Nguyen Trung M, Diener M, Fiedler D, et al. Investigation of a potential electrogenic transport-system for *myo*-inositol in the small intestine of laying hens. *Br Poult Sci* 2022;**63**:91-7. https://doi.org/10.1080/00071668.2021.1958301
12. Saenz JS, Kurz A, Ruczizka U, Bünger M, Dippel M, Nagl V, et al. Metaproteomics reveals alteration of the gut microbiome in weaned piglets due to the ingestion of the mycotoxins deoxynivalenol and zearalenone. *Toxins* 2021;**13**:583. https://doi.org/10.3390/toxins13080583
13. Cheng K, Ning Z, Zhang X, Li L, Liao B, Mayne J, et al. MetaLab: an automated pipeline for metaproteomic data analysis. *Microbiome* 2017;**5:**157. https://doi.org/10.1186/s40168-017-0375-2
14. Gurbich TA, Almeida A, Beracochea M, Burdett T, Burgin J, Cochrane G, et al. MGnify Genomes: A resource for biome-specific microbial genome catalogues. *J Mol Biol* 2023;**435**:168016. https://doi.org/10.1016/j.jmb.2023.168016
15. Cox J, Hein MY, Luber CA, Paron I, Nagaraj N, Mann M. Accurate proteome-wide label-free quantification by delayed normalization and maximal peptide ratio extraction, termed MaxLFQ. *Mol Cell Proteomics MCP* 2014;**13**:2513-26. https://doi.org/10.1074/mcp.M113.031591
16. Bray JR, Curtis JT. An Ordination of the upland forest communities of Southern Wisconsin. *Ecol Monogr* 1957;**27**:325-49. https://doi.org/10.2307/1942268
17. Oksanen J, Simpson GL, Blanchet FG, Kindt R, Legendre P, Minchin PR, et al. Vegan: Community Ecology Package. 2026.
18. Anderson MJ. A new method for non-parametric multivariate analysis of variance. *Austral Ecol* 2001;**26**:32-46. https://doi.org/10.1111/j.1442-9993.2001.01070.pp.x
19. Anderson MJ. Distance-based tests for homogeneity of multivariate dispersions. *Biometrics* 2006;**62**:245-53. https://doi.org/10.1111/j.1541-0420.2005.00440.x

20. Xiong Y, Mueller RS, Feng S, Guo X, Pan C. Proteomic stable isotope probing with an upgraded Sipros algorithm for improved identification and quantification of isotopically labeled proteins. *Microbiome* 2024;**12**:148. https://doi.org/10.1186/s40168-024-01866-1
21. Mellacheruvu D, Wright Z, Couzens AL, Lambert J-P, St-Denis NA, Li T, et al. The CRAPome: a contaminant repository for affinity purification–mass spectrometry data. *Nat Methods* 2013;**10**:730-36. https://doi.org/10.1038/nmeth.2557
22. Richardson L, Allen B, Baldi G, Beracochea M, Bileschi ML, Burdett T, et al. MGnify: the microbiome sequence data analysis resource in 2023. *Nucleic Acids Res* 2023;**51**:D753-59. https://doi.org/10.1093/nar/gkac1080
23. Hernández-Plaza A, Szklarczyk D, Botas J, Cantalapiedra CP, Giner-Lamia J, Mende DR, et al. eggNOG 6.0: enabling comparative genomics across 12535 organisms. *Nucleic Acids Res* 2023;**51**:D389-94. https://doi.org/10.1093/nar/gkac1022
24. Galperin MY, Vera Alvarez R, Karamycheva S, Makarova KS, Wolf YI, Landsman D, et al. COG database update 2024. *Nucleic Acids Res* 2025;**53**:D356-63. https://doi.org/10.1093/nar/gkae983
25. Kanehisa M, Furumichi M, Sato Y, Matsuura Y, Ishiguro-Watanabe M. KEGG: biological systems database as a model of the real world. *Nucleic Acids Res* 2025;**53**:D672-77. https://doi.org/10.1093/nar/gkae909
26. McDonald AG, Boyce S, Tipton KF. ExplorEnz: the primary source of the IUBMB enzyme list. *Nucleic Acids Res* 2009;**37**:D593-97. https://doi.org/10.1093/nar/gkn582
27. Paysan-Lafosse T, Andreeva A, Blum M, Chuguransky SR, Grego T, Pinto BL, et al. The Pfam protein families database: embracing AI/ML. *Nucleic Acids Res* 2025;**53**:D523-34. https://doi.org/10.1093/nar/gkae997
28. Darzi Y, Letunic I, Bork P, Yamada T. iPath3.0: interactive pathways explorer v3. *Nucleic Acids Res* 2018;**46**:W510-13. https://doi.org/10.1093/nar/gky299

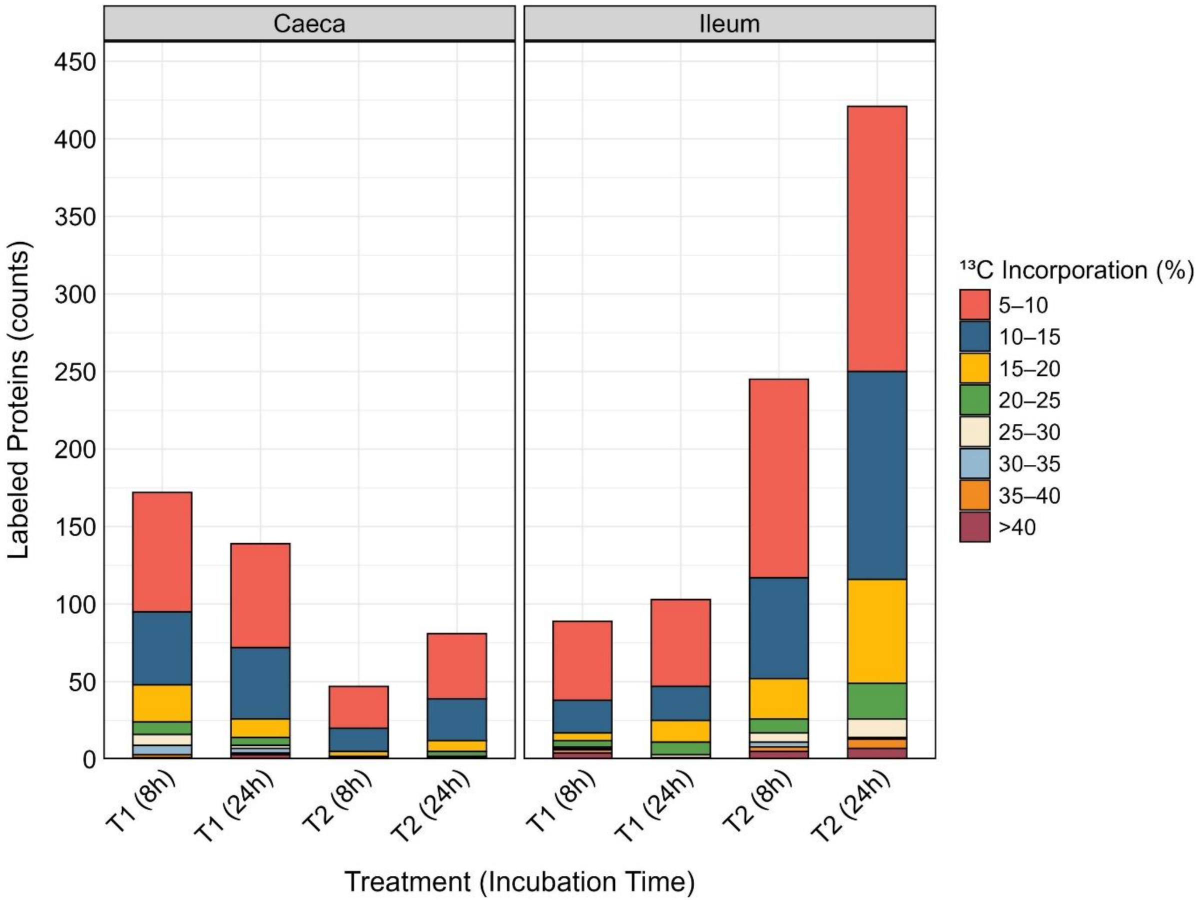

Fig. S1. Effect of treatment observed in previous trial. Number of labelled proteins detected in previous trial which counts on y-axis. The counts are without global protein removal and only after 5% ^{13}C-incorporation threshold. X-axis has information about the samples. T1 indicates treatment 1 which had no pre-incubation, and T2 indicates treatment 2 which had 24 h pre-incubation step. Time denoted inside bracket indicates the incubation time of the sample after treatment.

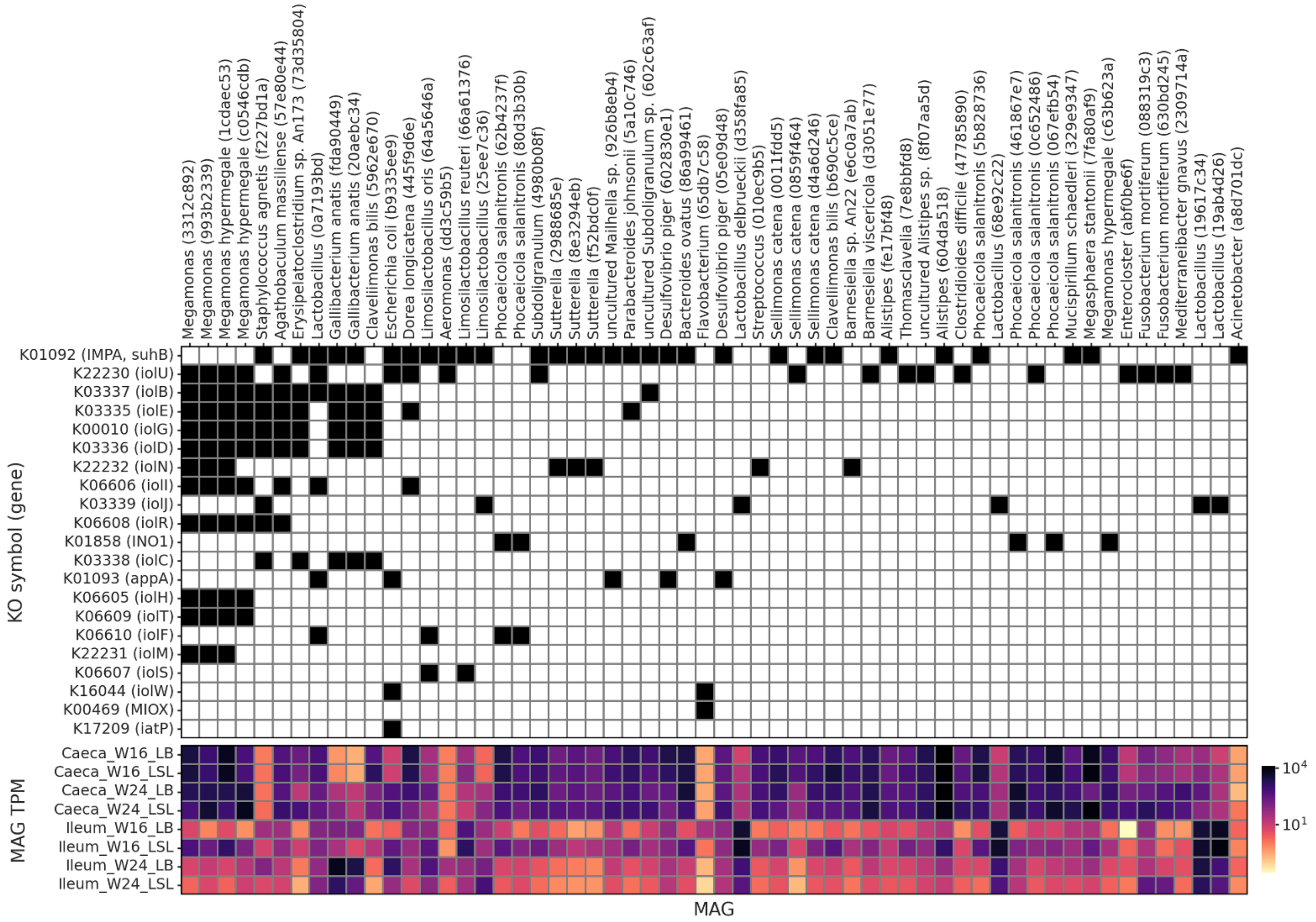

Fig. S2. Distribution of MI catabolic genes across MAGs from an independent laying hen microbiome dataset. The upper panel shows the presence or absence of genes associated with MI catabolism (including *iol* genes) in high-quality MAGs. MAGs are labelled according to their taxonomic assignment and unique genome identifier. The lower panel shows MAG abundances expressed as transcripts per million (TPM) across intestinal section, hen strain, and sampling time. Figure adapted from Yergalyiev et al. (2026) [1].

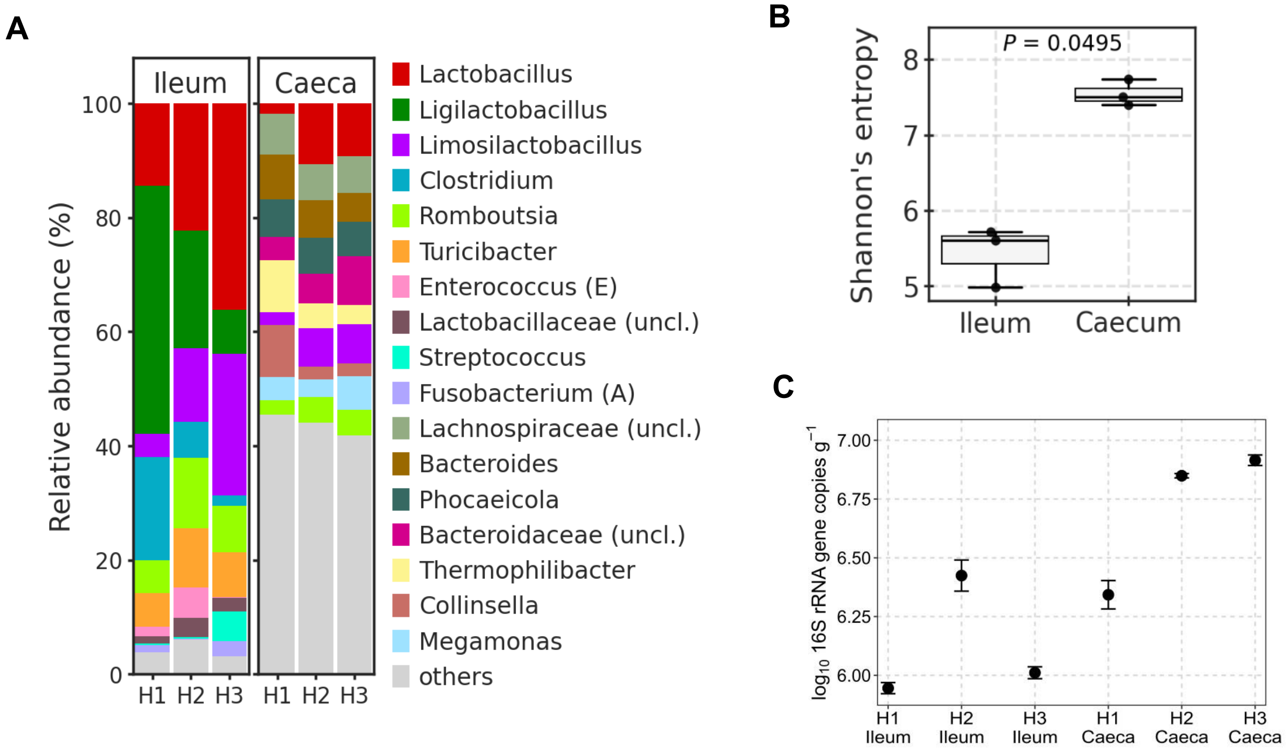

Fig. S3. Initial microbial community composition, alpha diversity, and bacterial 16S rRNA gene copy number across intestinal sections and hens. (A) Relative abundance of bacterial genera based on 16S rRNA gene amplicon sequencing. Y-axis indicates the relative abundance of each genus and X-axis indicates individual hens. Panels are separated by the section i.e. ileum or caeca. Only the top 10 most abundant genera are shown and all remaining genera are grouped as "Others". (B) Alpha diversity based on 16S rRNA gene amplicon sequencing. The X-axis indicates the intestinal section and the Y-axis indicates Shannon's diversity index. Each dot represents an individual hen, and error bars represent ± standard deviation (N=3). (C) Quantification of bacterial 16S rRNA gene copies in intestinal samples by quantitative PCR (qPCR). The Y-axis indicates log10-transformed bacterial 16S rRNA gene copies per gram of sample, and the X-axis indicates individual samples from each hen and intestinal section. Each point represents the mean value of six technical qPCR replicates, and error bars represent the standard deviation among technical replicates.

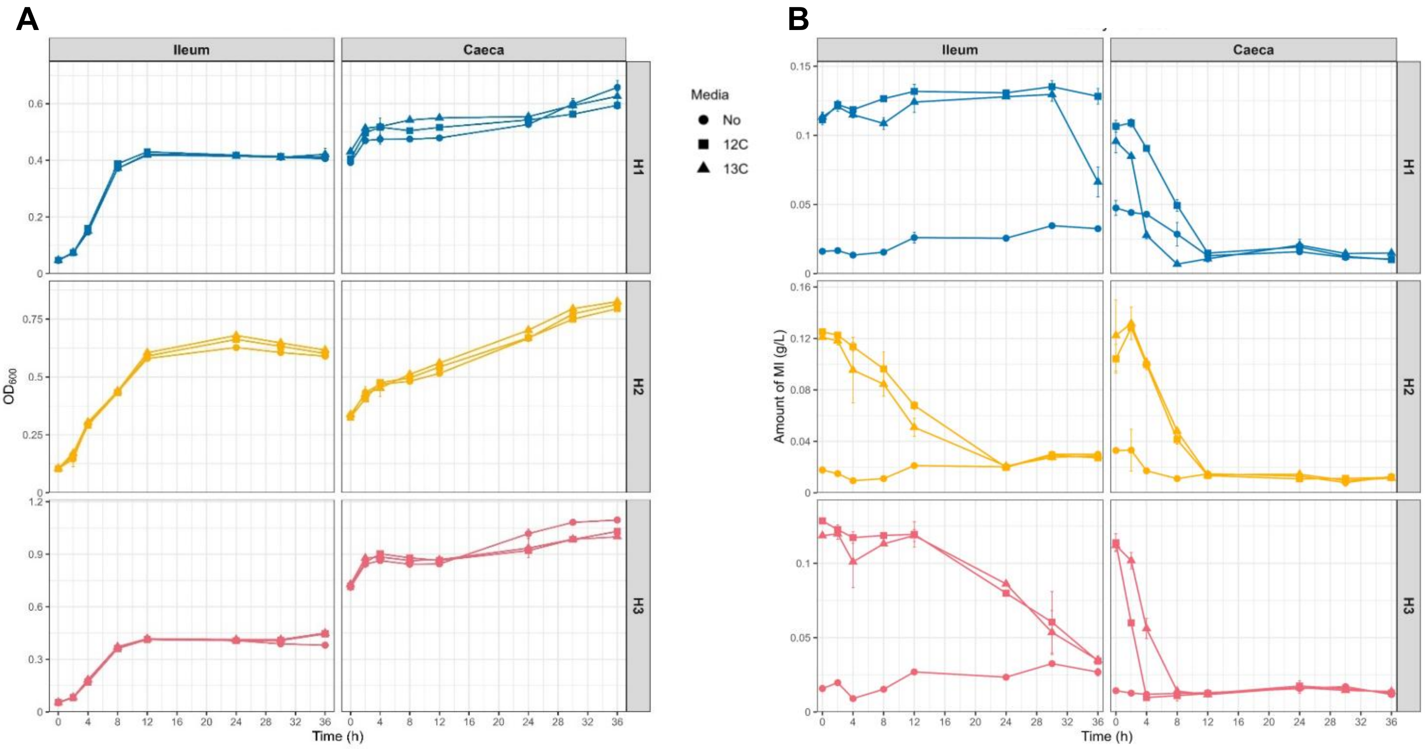

Fig. S4. Sample specific growth and MI depletion curve. (A) Sample specific growth curve; (B) Sample specific MI depletion curve. (A,B) Vertical panel indicates the sections. Horizontal panel indicates the hen. Different shapes of the points indicate the medium. Error bars represent the ± standard deviation (N=3).

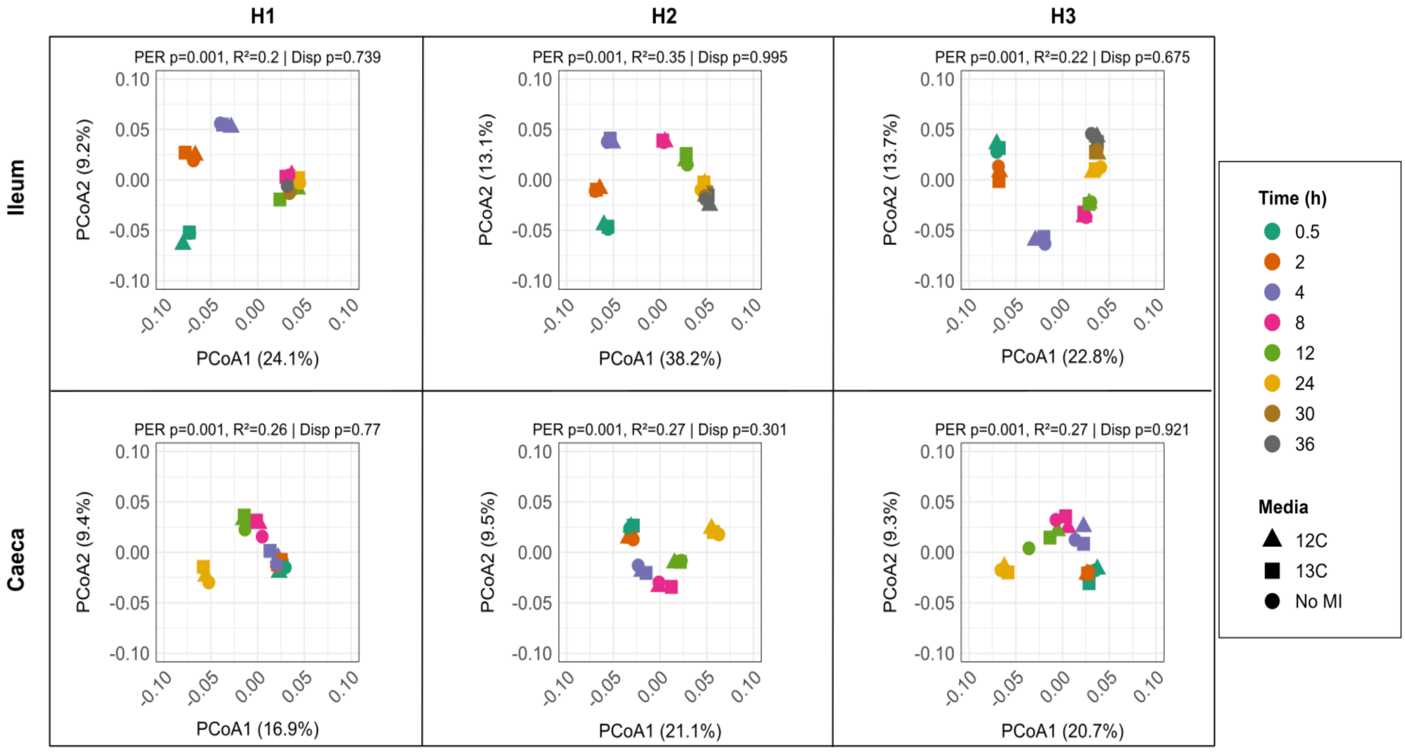

Fig. S5. Principal Coordinates Analysis (PCoA) based on taxonomic profile inferred from metaproteomic data. Each point represents a centroid of triplicate biological sample. Shape indicates the medium, colour indicates incubation time (hours). Axes represent the first two principal coordinates, with percentage of total variation explained indicated in parentheses. H1, H2, H3 indicate hen 1, hen 2 or hen 3. PER = PERMANOVA; Disp = Dispersion.

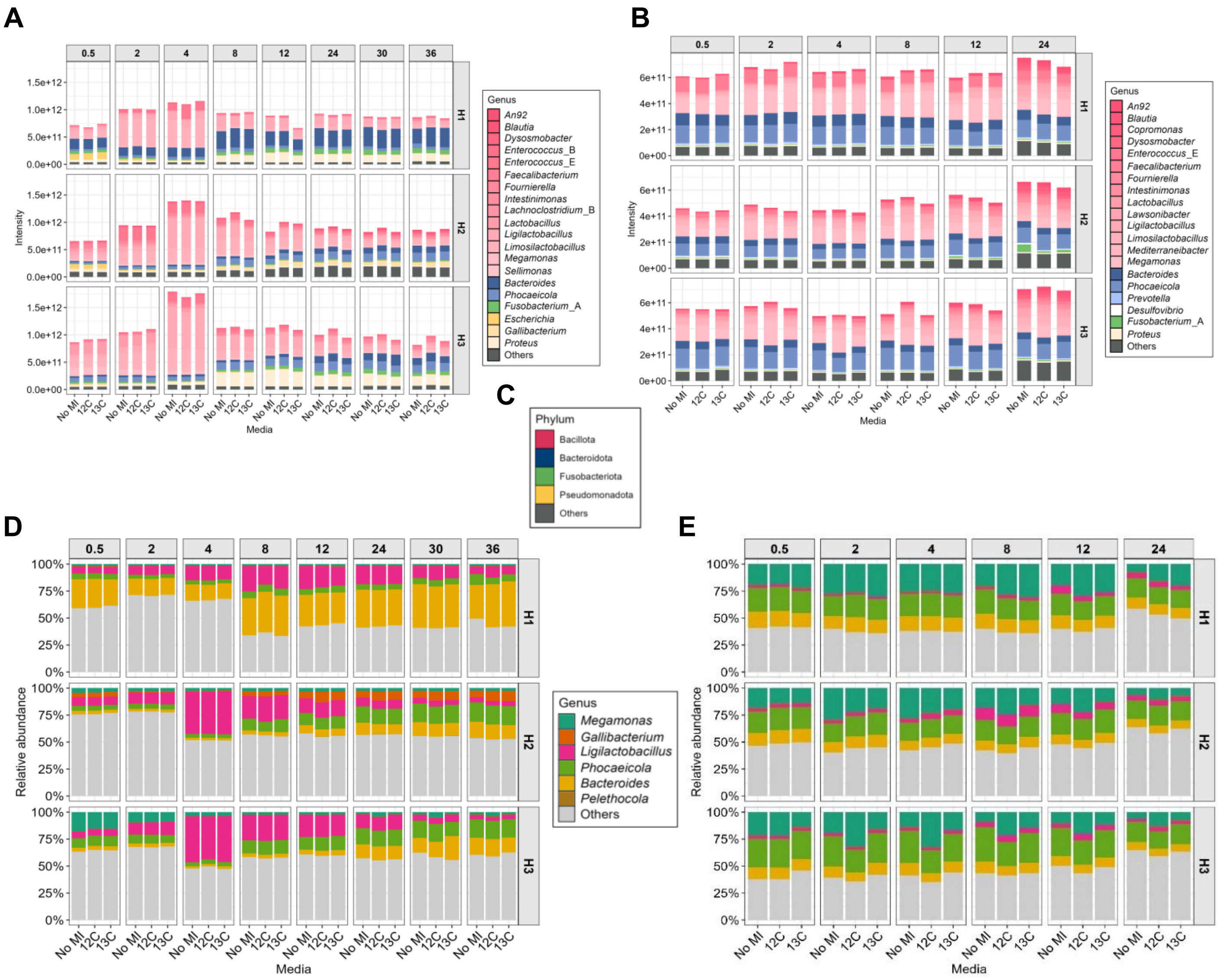

Fig. S6. Metaproteomics derived microbial community composition at genus and phylum levels across intestinal sections and media. (A,D) Ileum (B, E) Caeca; (A,B) Bar plots showing the intensity of the top 20 genera per intestinal section across media conditions ("No MI", "12C", "13C"). Genera outside the top 20 are grouped as "Others". Media is on X-axis and intensity is on Y-axis. Each row represents individual hen and each column represents incubation time; (C) Phylum legend: Colours indicate phylum identity; within each phylum, gradients distinguish individual genera in (A) and (B).

(D,E) Bar plots indicating relative abundance of selected genera per intestinal section across media conditions ("No MI", "12C", "13C"). Genera outside the selected are grouped as "Others". Media is on X-axis and relative abundance is on Y-axis. Each row represents individual hen, and each column represents incubation time.

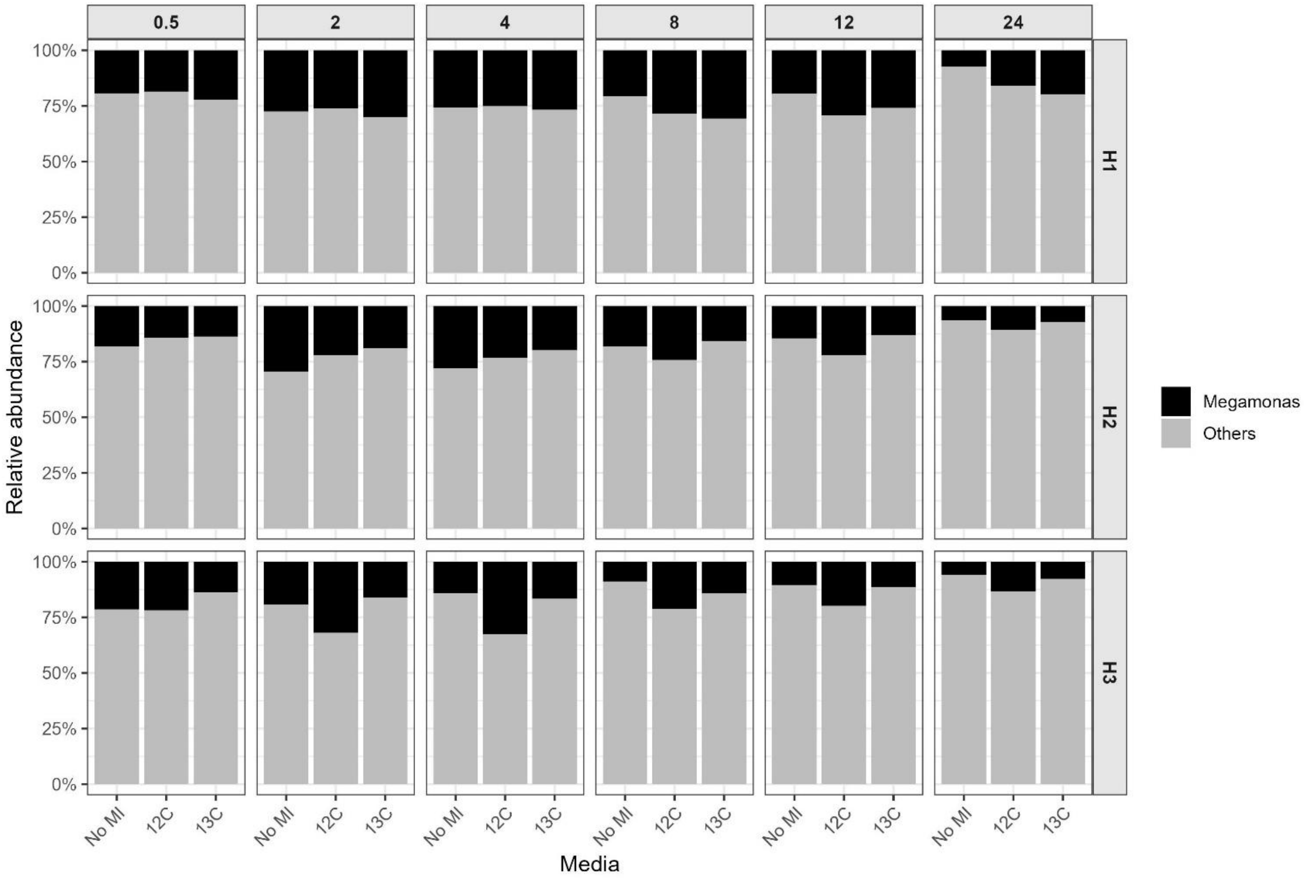

Fig. S7. Relative abundance of *Megamonas* across media and time in caeca. X-axis indicates the relative abundance (%) and y-axis indicates the media. Vertical panel denotes the incubation time (in h) and horizontal panel indicates the hen.

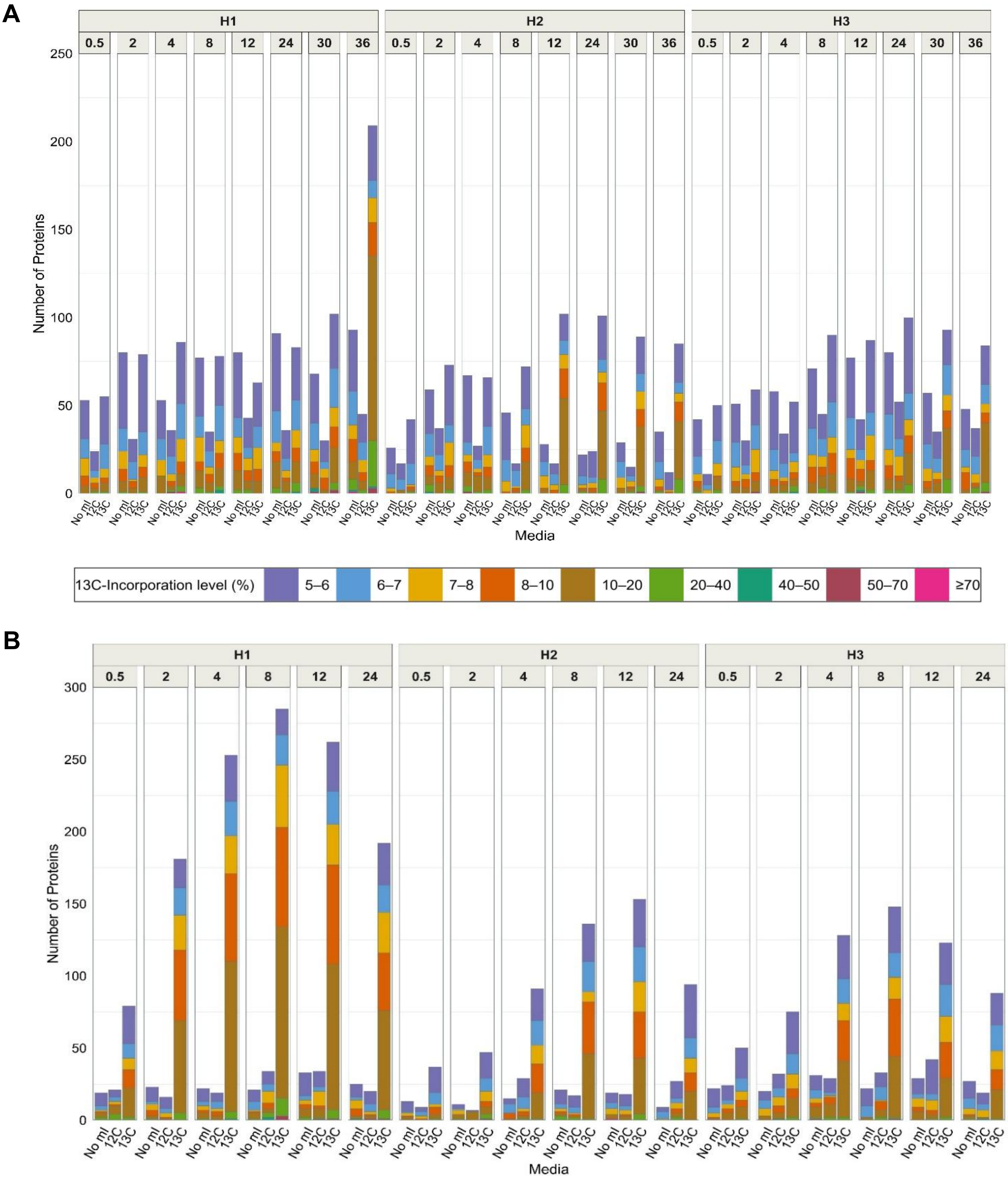

Fig. S8. Distribution of isotopically labelled proteins across intestinal sections and hens before global control removal. Stacked bar plots showing the number of isotopically labelled proteins detected per sample in the ileum (A) and caeca (B) across hens (H1-H3), time points, and growth media. Counts represent proteins retained after replicate filtering and enrichment thresholding before global control removal. Proteins are grouped by average enrichment level (%), and colour coding indicates the enrichment categories.

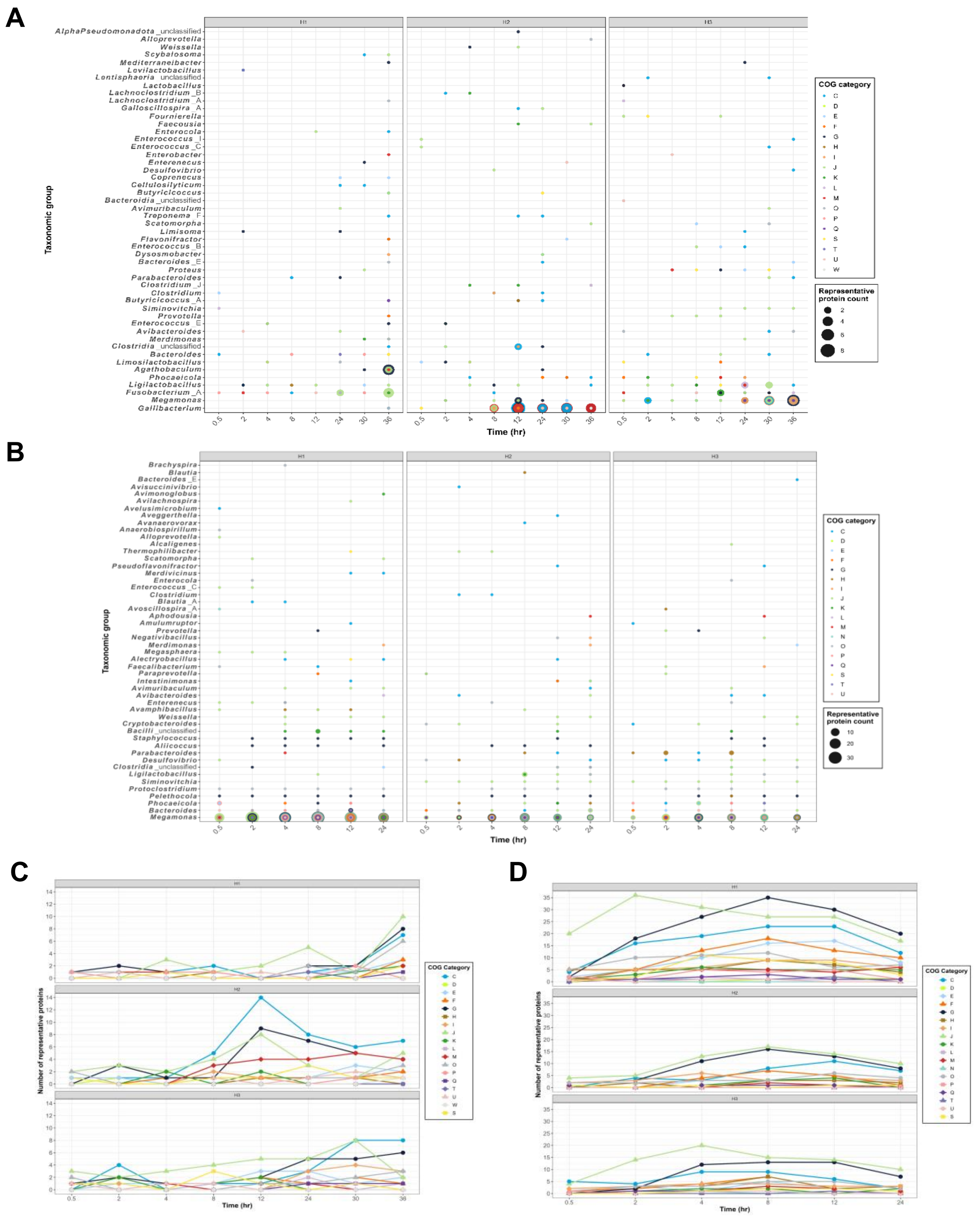

Fig. S9. Functional profiles and temporal dynamics of representative labelled proteins in the ileum and caeca with top 50 taxa and full COG dynamics. (A,C) Ileum; (B,D) Caeca. (A,B) Bubble plots showing the distribution of representative labelled proteins across COG categories over time. Bubble size reflects the number of representative proteins assigned to each COG category. Vertical panels correspond to the three hens. (C,D) Temporal trajectories of all COG categories based on representative labelled proteins within each section. Lines indicate changes in protein counts across incubation time. Horizontal panels correspond to the three hens.

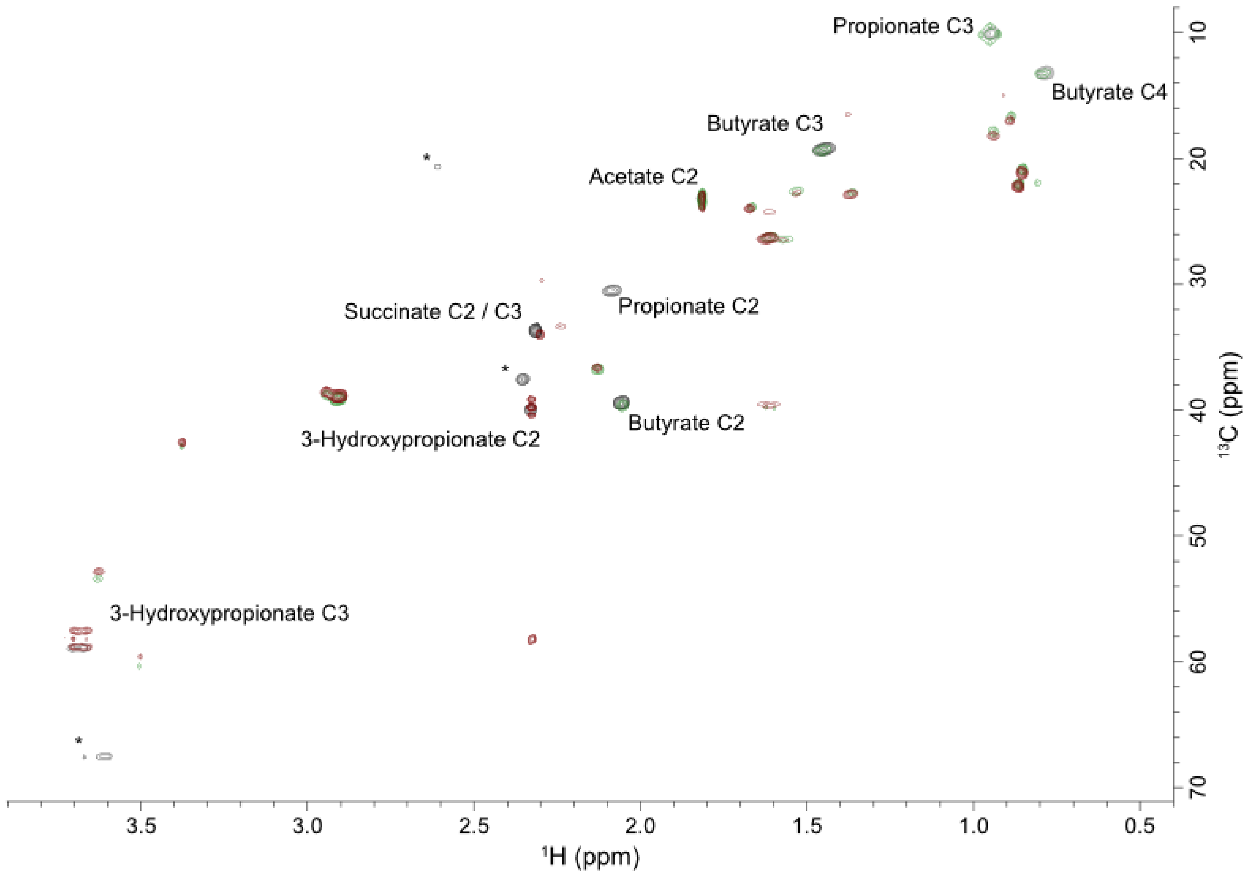

Fig. S10. Carbon NMR spectra of reference metabolites. Overlay of the alkyl region of 2D heteronuclear HSQC experiments of reference compounds (black: CH_2 groups, grey: CH_3 groups) and two samples of the study. Red: Ileum sample of H3 at 36h, containing 3-hydroxypropionate, acetate and succinate (propionate not visible at that threshold due to ^{13}C induced peak splitting). Green: Caeca sample of H3 at 36h, containing acetate, butyrate and propionate (H_2/C_2 signal of propionate not visible due to ^{13}C induced peak splitting). *: signals of the 3-hydroxypropionate reference compound (purity 30%) other than 3-hydroxypropionate. Chemical shifts due to slight pH deviations between different samples. Non-annotated red / green peaks stem from matrix components.

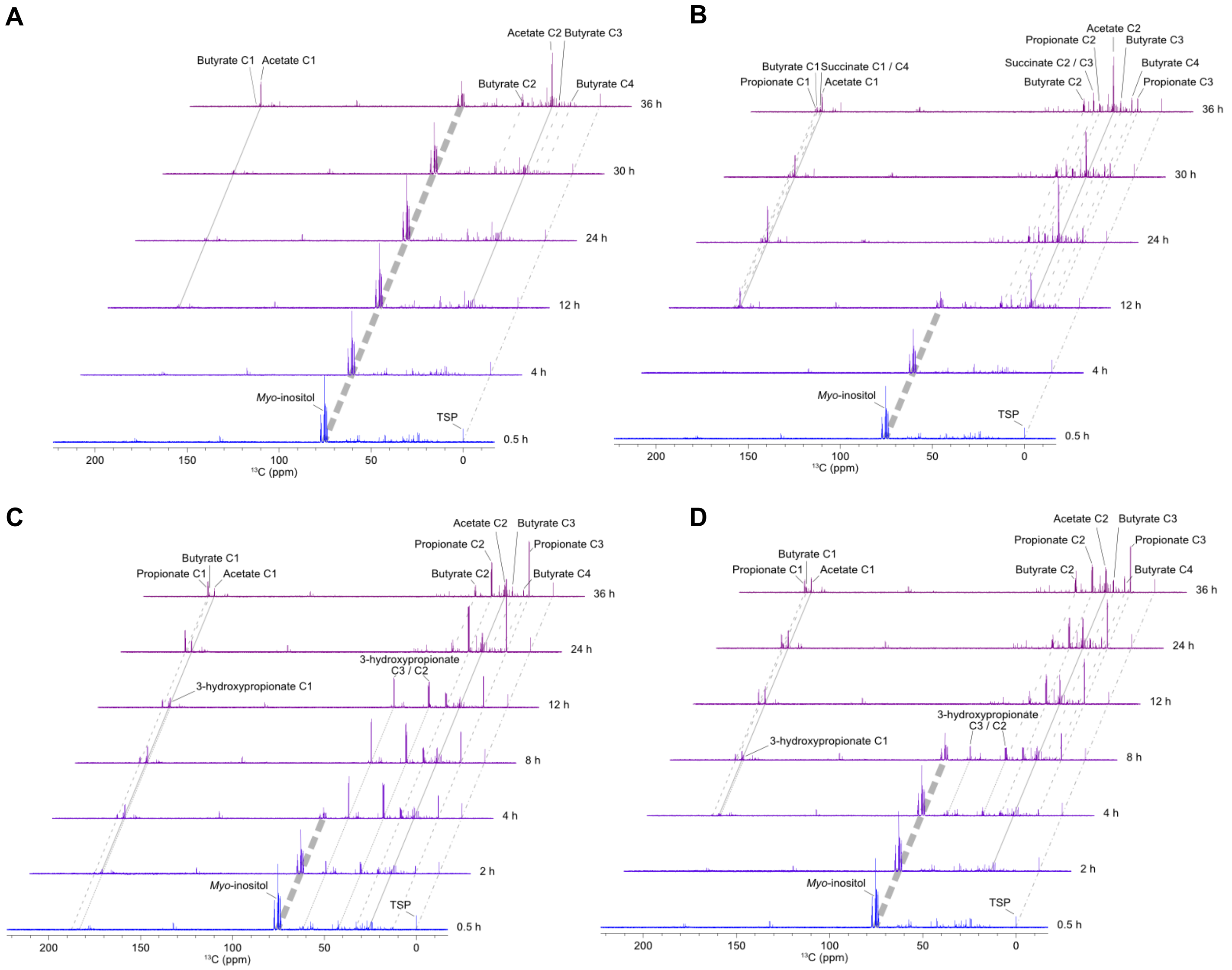

Fig. S11. Carbon NMR spectra of H1 and H2. Overlaid ^{13}C-NMR spectra across the time course showing the decline of the ^{13}C-*myo*-inositol resonance and the emergence of ^{13}C-labelled metabolites. (A) H1 Ileum; (B) H2 Ileum; (C) H1 Caeca; (D) H2 Caeca. The TSP internal standard remains constant and is included for chemical-shift reference and confirms consistent spectral calibration across measurements.

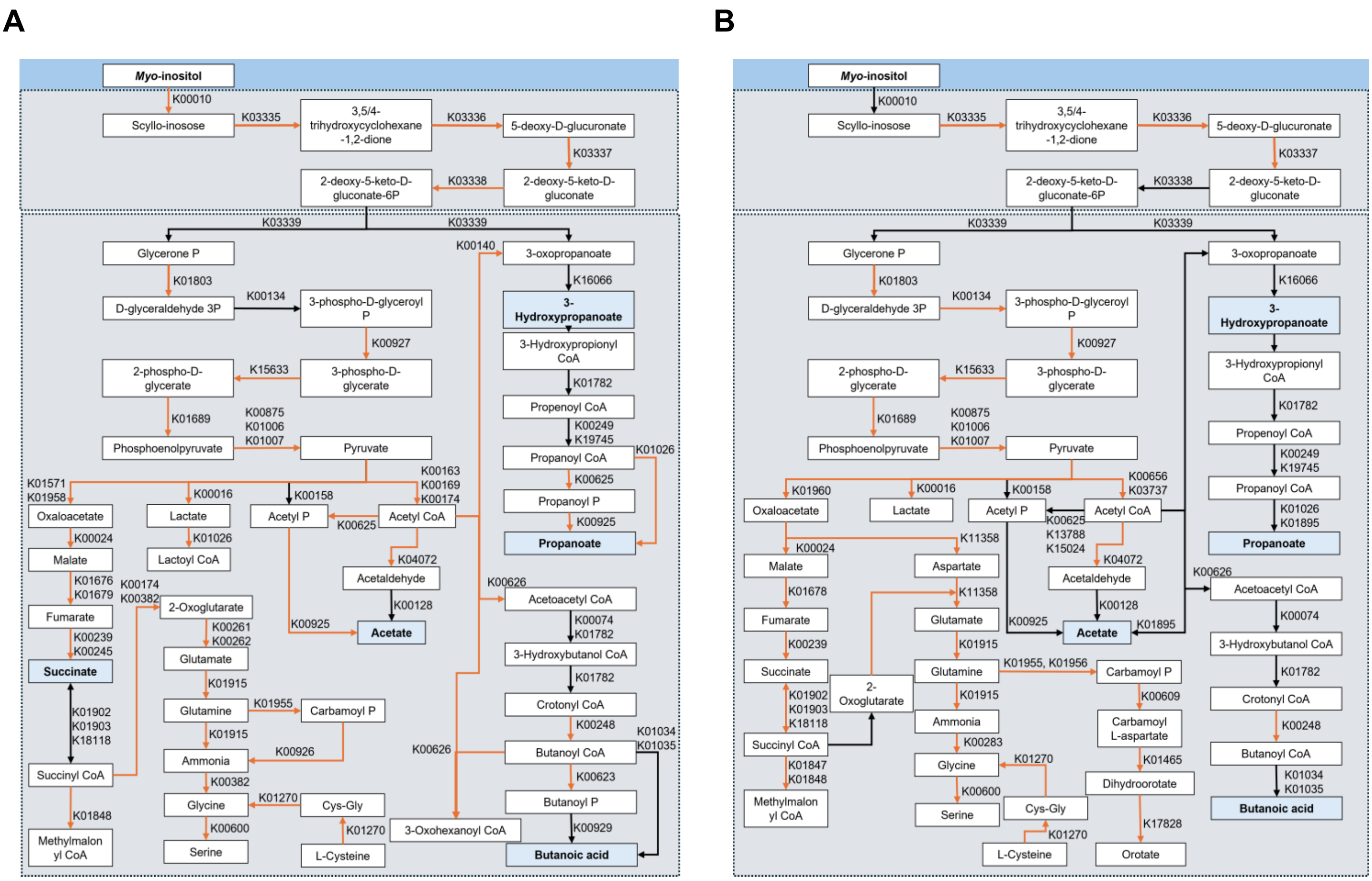

Fig. S12. Integrated reconstruction of microbial *myo*-inositol degradation in the hen gut. (A) Ileum; (B) Caeca; Orange lines indicate the labelled enzymes whereas black lines indicate the unlabelled enzymes. Highlighted blue boxes indicate the detection of those metabolites as labelled in ^{13}C-NMR.
